# Walking in circles: linking high- and low-level parameter scaling of visually guided and spontaneous turning behaviour

**DOI:** 10.1242/jeb.252608

**Published:** 2026-10-06

**Authors:** Merit Meschenmoser, Volker Dürr

**Affiliations:** ^1^ Biological Cybernetics, Faculty of Biology, Bielefeld University, 33615 Bielefeld, Germany; ^2^ Center for Cognitive Interaction Technology, Bielefeld University, 33615 Bielefeld, Germany

**Keywords:** Turning, Kinematics, Locomotion, Visual orientation, Leg movement, Spontaneous behaviour

## Abstract

The ability to change one's current heading, i.e. to turn, is essential for all walking animals. While several studies have addressed how leg movement or inter-leg coordination may change during turning, relatively little is known about how turning-related changes at the level of single-leg stepping (low level) scale with turn magnitude at the level of body trajectories (high level). Here, we used spontaneous and visually induced turns of unrestrained walking stick insects to test (i) how high-level parameters of unrestrained turning scale with low-level parameters of leg movement, and (ii) the effect of visual guidance on turning parameters. To this end, we used a step change in stationary landmark position in an open-field arena to constrain the timing and magnitude of target-directed turns. These visually guided turns were compared with spontaneous turns in an all-white condition without guiding landmarks. We show that visually induced turns were walked at a greater forward velocity and had fewer short steps than spontaneous turns. The scaling of turning responses was dominated by an increase in turning duration (factor 1.87) rather than turning speed (factor 1.32). Increased rotational velocity correlated with reduced forward velocity, though with flexible timing of both effects. These changes were accompanied by larger shifts in step direction, as well as an increased asymmetry of step types between inner and outer legs, suggesting a mix of distinct turning strategies that depend on overall turn angle. Future models on six-legged locomotion should thus consider the incorporation of more than one mechanism to govern turning.

## Teaser

**Summary:** Freely walking stick insects turn by a combination of scaled changes in step parameters and frequency of distinct step types.

## INTRODUCTION

In terrestrial locomotion, complex movement sequences as part of searching, climbing or turning behaviours require not only appropriate temporal coordination of several legs but also adaptation of spatial step parameters such as step length and direction (Ritzmann and Büschges, 2007; Cruse et al., 2009a; Büschges and Ache, 2025). With regard to turning and curve walking, behavioural and biomechanical evidence about ‘low-level’ step parameter changes in stick insects (Dürr and Ebeling, 2005; Cruse et al., 2009b; Gruhn et al., 2009), cockroaches (Franklin et al., 1981; Nye and Ritzmann, 1992; Jindrich and Full, 1999; Ridgel et al., 2007) and other insects (e.g. Strauss and Heisenberg, 1990; Frantsevich and Cruse, 2005; Zolotov et al., 1975) has been complemented by predominantly neural evidence on descending control of ‘high-level’ turning trajectories (Yang et al., 2024; Rayshubskiy et al., 2025; Cruz and Chiappe, 2023). Still, the direct link between low-level changes in leg movement and high-level changes in body trajectory is not well understood, particularly not in unrestrained locomotion.

Previously, turning in stick insects has been elicited visually through large-field motion cues (Dürr and Ebeling, 2005; Gruhn et al., 2009) or salient, stationary landmarks (Kalmus, 1937; Rosano and Webb, 2007; Zeng et al., 2020; Meschenmoser and Dürr, 2025; Bigge et al., 2026). However, with either stimulus type, it is unknown how turning scales with stimulus magnitude. For example, in order to cover twice the turn angle, animals could either turn for twice as long or turn twice as fast, or a combination of the two. Our experiments were designed to test for these alternatives, employing an instantaneous displacement of a visual target to elicit two different turn angles within a defined region of our setup.

While it is known that changes in leg movement parameters during turning can be related to the curvature of the walked path (e.g. Jander, 1985), adaptive control of (at least) 18 degrees of freedom allows insects to employ various strategies to walk the same curve. Nevertheless, current locomotion models typically implement only a single mechanism that governs turning. Recently, individual idiosyncrasies have been shown to shape straight walking behaviour in *Drosophila melanogaster* (Godesberg et al., 2024). However, this description of inter-individual variation is not yet fully integrated in an understanding of inter-trial variation, which is necessary to distinguish adaptive control of speed and heading from redundancy-related variability of movement. Here, we tested whether insects constrain this redundancy in a context-dependent manner by comparing visually guided with spontaneous, unguided turning.

Although stick insects have comparatively unspecialised legs, turning is thought to be initiated by strong changes in front leg stepping (Dürr and Ebeling, 2005) and reduced coupling to ipsilateral middle and rear legs (Dürr, 2005). Accordingly, the front legs have been proposed to be the primary target of visual descending information about how to change heading. Indeed, simulations involving active changes in front leg movement only, with middle and hind legs following passively, have reproduced similar turns to those performed by stick insects (Rosano and Webb, 2007). More generally, front legs show the largest degree of motor flexibility of all leg pairs, as reflected by their key function in near-range searching (Dürr, 2001; Berg et al., 2013; Dürr and Mesanovic, 2023), local probing (Bläsing and Cruse, 2004a) and targeted reaching (Schütz and Dürr, 2011). When studying step cycle parameter variation during unrestrained walking and climbing, Theunissen and Dürr (2013) found that step length distributions of all leg pairs were bimodal. Given the distinct properties of step cycles sampled from these two modes, they suggested that insects generate two distinct classes of step cycles: While ‘long steps’ were proposed to serve propulsion, ‘short steps’ were interpreted as correction steps in case of insufficient foothold. As short steps occurred more frequently on the edges of objects during climbing, they could also serve repetitive probing or exploration functions, as proposed for short steps before and during gap-crossing manoeuvres (Bläsing and Cruse, 2004a). Assuming an exploratory function of intermittent short steps, we expect the frequency of short steps to increase as spatial information about the environment becomes less reliable. Accordingly, we tested the hypothesis that the absence of visual orientation cues leads to increased exploratory effort, i.e. more short steps, to compensate for the lack of visual spatial cues.

Our experiments thus tested the effect of visual guidance on unrestrained turning responses, along with the effect of stimulus magnitude, i.e. the step change in target position. To this end, we used two displacement magnitudes (60 deg, 120 deg) and an all-white stimulus to compare visually guided and spontaneous turns of equal curvature. A stationary landmark with no displacement was included as a control for ‘straight walking’. Our analysis links two levels of description of turning behaviour: first, we analysed body axis kinematics, relating the timing and magnitude of forward translational and rotational velocities; second, we related these changes to step cycle parameters of the front legs, with a focus on step length and swing direction.

## MATERIALS AND METHODS

### Experimental animals and setup

We collected data from 52 adult, female stick insects of the species *Carausius morosus* (Sinéty, 1901). Of these, 20 stick insects were used in the preliminary experiment and 32 in the main experiment. Specimens were taken from a parthenogenetic breeding colony at Bielefeld University. The animals were kept on a 12 h:12 h light:dark cycle and fed *ad libitum* with blackberry leaves and Chinese cabbage.

Animals were placed manually into a circular open-field arena with a diameter of 120 cm, surrounded by a translucent screen of 20 cm height. Using external projectors, the screen was illuminated with white light from the outside. In most conditions, a black bar was projected onto the white screen, serving as a visual landmark. The bar was 20 deg wide as seen from the centre of the arena. Animals were free to walk on the black wooden arena floor. Top-view videos were collected at a frame rate of 50 Hz, using a custom-built camera gantry that was equipped with an infrared-sensitive digital video camera (Basler A602f-2 or A602fc-2, image size: 640×480 px) and a manual zoom lens (Computar, M6Z1212-3S). For a detailed setup description see Niemeier et al. (2021) and Meschenmoser and Dürr (2025).

### Experimental procedure

In trials with a landmark, animals were always placed in the arena opposite to and facing the landmark. To induce a sudden shift of landmark position, the experimenter manually triggered a step change in landmark position when an animal crossed the midline of the arena, using a wireless remote control (Kensington K72426EU). The magnitude of this landmark displacement varied among conditions and was set by a custom-written MATLAB script. In ‘all-white’ control trials without a landmark, animals were put in the arena opposite to where the black bar would have been in landmark trials (Fig. 1A). The person handling the animal was unaware of the order in which conditions were presented. Time stamps of the change in landmark position were saved in a text file. The video recording was terminated once an animal reached the arena wall or stopped walking.

**Fig. 1.**
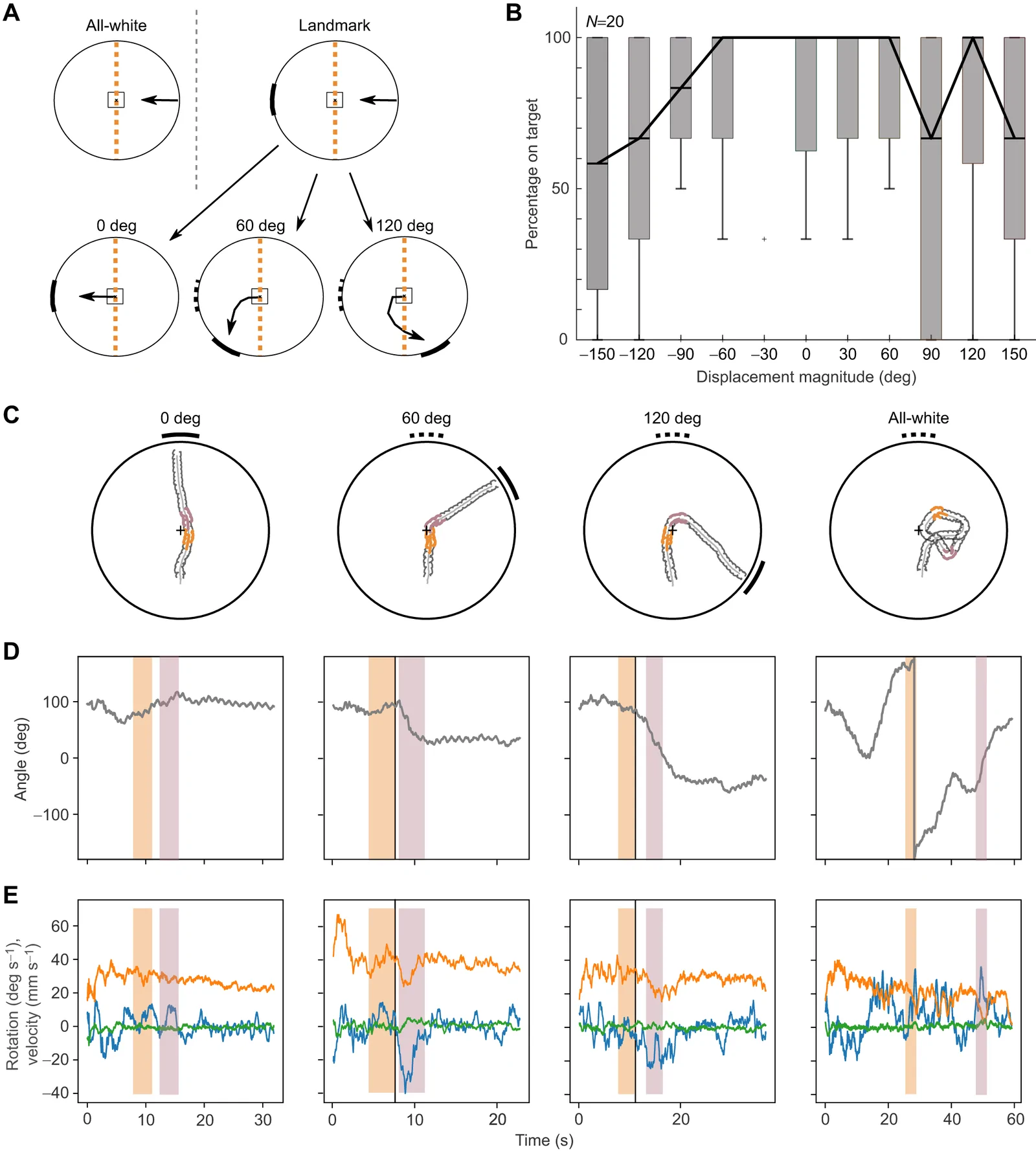
**Experimental design and example trials.** (A) Schematic drawing of the circular arena and experimental procedure. In landmark trials, the position of the landmark was changed as soon as the animal reached the arena centre. All-white trials served as controls. Solid black lines outside the arena represent the landmark position and width; dotted black lines in the second row show the original position of the landmark before the displacement. The black arrow in the upper row shows the start orientation of the animal. Arrows in the second row show the hypothesised orientation response of the animal. The black cross and the dashed orange line highlight the centre point and midline of the arena, respectively. (B) Boxplots show the likelihood of an animal being ‘on target’ for different displacement magnitudes, with *N*=20 means per animal, from 3 trials per animal and displacement. Boxes show inter-quartile range (IQR), with horizontal line showing the median, whiskers show minimum and maximum. (C–E) Random example trials from the same animal (Movies 1–4). (C) Top view of the circular arena. The light grey trace shows the trajectory of the metathorax marker, while the dark grey lines are the trajectories of the tarsal tips of both front legs. Bold, coloured parts of the trajectories highlight the two episodes, A and B (straight and curved walking, respectively; see Fig. 2). (D) Body orientation throughout a trial. The vertical black lines indicate when the landmark was displaced in 60 deg and 120 deg trials. Body orientation angles above or below ±180 deg were converted to angles within the ±180 deg range. Coloured shading marks the two episodes, A and B. (E) Three movement components of the centre of mass on a planar surface: rotational (blue), forward (orange) and sideward translational (green) velocity. Coloured shading marks the two episodes, A and B.

**Fig. 2.**
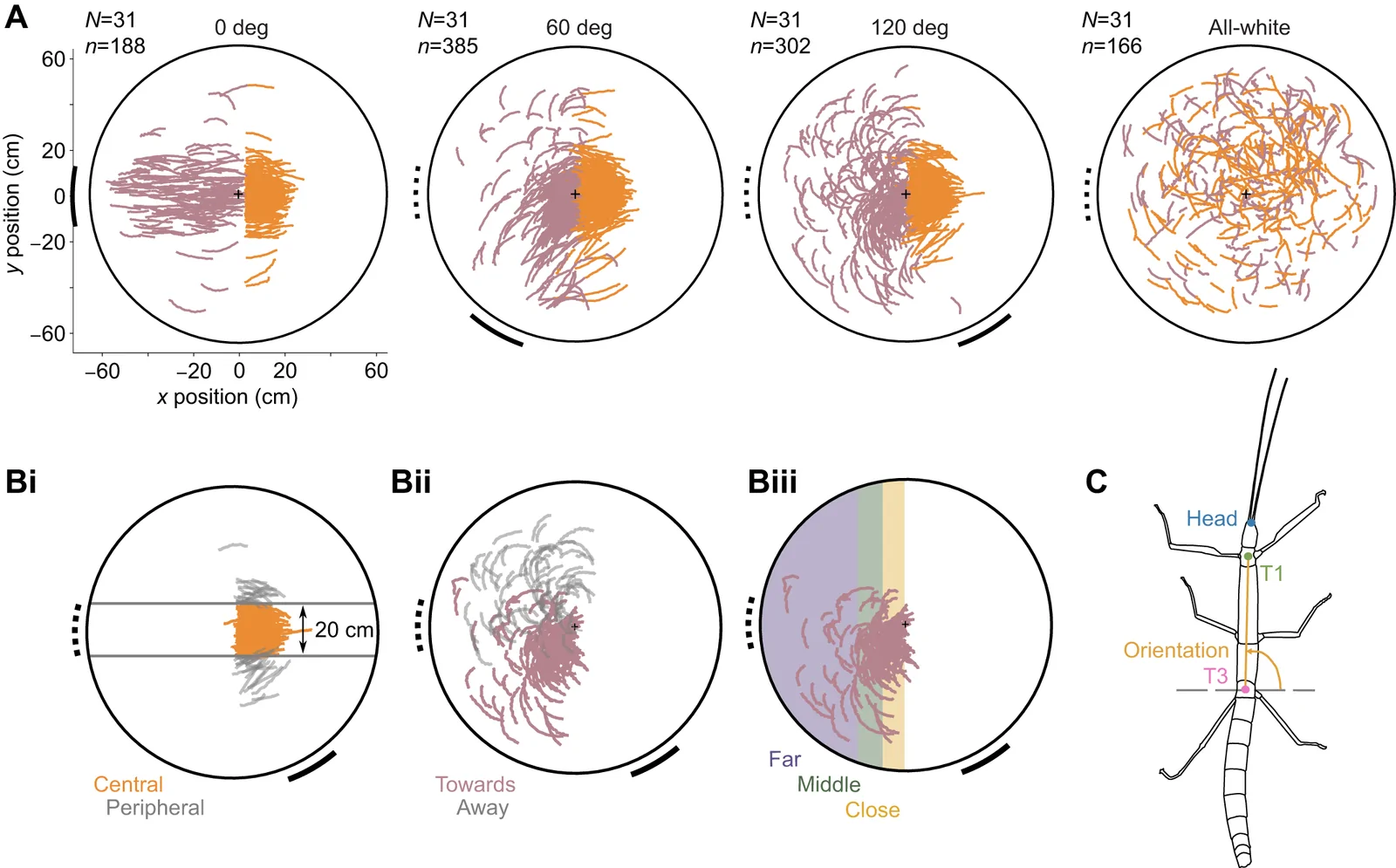
**Episode overview and categorisation of turning responses.** (A) Top view of the circular arena. Solid black lines outside the circle show the width and position of the black bar presented; dotted black lines show the first position of the black bar before it was displaced in 60 deg and 120 deg trials. In the all-white condition, the dotted line indicates the start orientation of the animal, as no black bar was present at any time. The black cross indicates the midpoint of the arena. Circular plots show the prothorax (T1) positions during two episodes of each ‘on-target’ trial. For the all-white condition, all trials are shown. The two episodes were defined as ‘pre-turning’ (episode A, orange) and ‘turning’ (episode B, pink). The number of animals (*N*) and plotted trials (*n*) is shown in the upper left of each panel. Note that the spread of all trajectories does not reflect the likelihood of animal position in the arena, as it strongly overrates peripheral trajectories (see Fig. 4A). (B) Schematic drawings of the categorisation process of turning responses according to their spatial occurrence. (Bi) Only ‘central’ trials in which T1 stayed within a corridor of ±10 cm relative to the midline at the end of episode A (orange) were used for further categorisation. (Bii) Depending on turning direction during episode B (pink), trials were categorised as ‘away’ or ‘towards’. (Biii) ‘Towards’ trials were further categorised as close (yellow), middle (green) and far (purple) turns, depending on the location of episode B (pink). (C) Schematic drawing of a stick insect. Coloured dots show the thorax features that were used for calculating the body axis orientation of an animal.

Trials were scored as ‘at-wall’ and ‘on-target’ trials. An animal was considered ‘at wall’ if its head was less than 7 cm away from the wall. To be ‘on target’, animals had to be ‘at wall’ in the target range. This was defined as the target width plus a tolerance of 5 deg on either side of the target. As there was no target present in the all-white condition, trials could be ‘at wall’ but not ‘on target’.

### Preliminary experiment

A preliminary experiment was carried out to determine the reliability of target approach for different magnitudes of landmark displacement. For each trial, we noted whether or not the animal reached the target. No videos were recorded. Our aim was to decide on two magnitudes which were considerably different while ensuring a high probability of approaching the target. To this end, we used 10 different displacement magnitudes and tested them in both clockwise and anti-clockwise directions (±30 deg, ±60 deg, ±90 deg, ±120 deg, ±150 deg). Judging from a microcomputed tomography (microCT) scan, we estimated the azimuthal range of visual field of each *C. morosus* compound eye to be almost hemispherical, with a mediad rotation of about 11 deg (M.M. and V.D., unpublished observations). Assuming interommatidial angles of 5 deg to 6 deg (Jander and Volk-Heinrichs, 1970), we expected animals to see the 20 deg landmark even after a displacement by 150 deg. Trials with a stationary landmark (0 deg) were used as a control. We used 20 animals and repeated each condition three times per animal in a pseudo-randomised manner, resulting in 33 single trials per animal. The results showed that the median likelihood to be ‘on target’ decreased at displacement angles beyond ±60 deg, but was never less than 60% (Fig. 1B).

### Experiment

Based on the results of our preliminary experiment, we decided on ±60 deg and ±120 deg as displacement magnitudes (Fig. 1A). This choice covered a large portion of the visual field and allowed for simple assessment of linear scaling of response parameters with displacement magnitude.

Data were collected in sets of five trials consisting of one 0 deg trial, two ±60 deg trials and two ±120 deg trials (one for each direction). The order in which the conditions were presented was randomised within each set. The starting orientation (90 deg or 180 deg) of the trial was randomised across all trials, including all-white trials, to exclude confounding, setup-specific effects. Experiments were done in two cohorts which differed slightly in the number of all-white trials and the number of sets. The first cohort experienced one all-white trial after two sets, whereas the second cohort experienced one all-white trial after every set. Additionally, we decreased the number of sets from 8 in the first cohort to 7 in the second cohort. This resulted in 44 trials per animal for the first cohort and 42 trials for the second cohort, respectively. In total, we collected 1252 trials from 32 animals. One animal was excluded from the analysis as it only contributed two at-wall trials to the dataset. The number of animal means used in the analysis varied between 26 and 31, as not every animal contributed data points for all response categories. Figure captions state the number of animals included.

### Markerless tracking and categorisation of trials and episodes

Data processing and analysis were done using Python 3.9.13 (van Rossum and Drake, 2009) and MATLAB 2022b (Natick, MA, USA; https://www.mathworks.com). ChatGPT 4o mini (OpenAI) was used to aid the development of code for data analysis. Artificially generated code was reviewed, edited, augmented and validated by the authors.

Digital videos were analysed so as to reconstruct body and leg positions, body axis orientation and head position. We used DeepLabCut (Mathis et al., 2018; Nath et al., 2019; Arent et al., 2021) to track several features on the body and the legs of the animal. Tracked features along the body axis were the midpoint between the two hindlegs on the metathorax (T3), the midpoint between the two middle legs on the mesothorax (T2), the midpoint between the two forelegs on the prothorax (T1) and the tip of the head (Hd). Additionally, we tracked four features per leg: the coxa–trochanter joint (CTr), the femur–tibia joint (FTi), the tibia–tarsus joint (TiTa) and the distal tip of the tarsus (Tars) (see Fig. S3). Only frames with a mean confidence rating above 95% across all features were used for further analysis. A median filter (width=3 frames) and a Gaussian kernel (full width at half maximum=5 frames; σ=2.12, kernel width=15 frames) were used to smooth feature trajectories. The body orientation was determined as the angle between the vectors T3→T1 and (0, 1)^T^ (see Fig. 2C).

A trial started when the animal no longer had contact with the experimenters' hand and ended either upon reaching the arena wall or if the animal stopped. Stopping was defined as the instant when forward velocity dropped below 1.0 mm s^−1^ for at least 1.5 s. For each trial, two episodes were compared, each one lasting 3 s. Episode A started 3 s before the change in landmark position. In 0 deg control trials, it started 3 s before the animal crossed the midline of the arena. In all-white trials, it started halfway through the trial. Episode B was defined as the 3 s interval centred on the maximum mean rotational velocity, i.e. the instant of strongest turning (Fig. 2A).

Only ‘on-target’ trials were used for further analysis of landmark trials. The number of animals varied for certain parts of the analysis due to the fact that not all animals were ‘on target’ in all conditions (as indicated in respective figures). All quantitative analyses and statistics were carried out on per-animal means. If not stated otherwise, we used a Friedman test and *post hoc* Wilcoxon signed-rank tests for matched pairs for statistical comparisons.

### Data analysis at the trajectory level

Kernel density estimates were calculated using the *gaussian_kde* function from *scipy.stats* with a 120 cm×120 cm grid and a grid resolution of 1 cm×1 cm. To allow for a comparison between different conditions, density estimates were normalised by the maximum density across all stimuli.

The overall curvature (or tortuosity) of a trial was calculated as the walked distance divided by the length of the shortest possible path, i.e. a straight line. Rotational velocities were calculated for ‘on-target’ trials only, using the filtered T1 and T3 positions (Sawitzky–Golay filter: polynomial order 1, window size 25 frames). As trajectories varied strongly, particularly in 120 deg trials, we categorised turning responses according to the location and direction of maximum turning to facilitate comparison between conditions (Fig. 2B). To exclude ‘peripheral’ trials in which animals left a straight corridor before landmark displacement, only ‘central’ trials in which the prothorax, T1, remained within 10 cm either side of the midline at the end of episode A were used for further categorisation (Fig. 2Bi). To account for turning direction, all trials in which the direction of rotation during episode B was directed away from the second landmark position were categorised as ‘away’ turns and not considered further (Fig. 2Bii). To account for latency of turning, ‘towards’ trials were further categorised according to the location of T1 at the start of episode B. If T1 remained within 10 cm of the arena centre, the trial was categorised as a ‘close’ turn. Between 10 and 20 cm from the arena centre, it was categorised as a ‘middle’ turn, and everything beyond 20 cm was considered a ‘far’ turn (Fig. 2Biii). For comparison of maximum rotational velocity, timing of maximum rotational velocity and duration of turning, we used ‘close turns’ only. As forward velocity decreased towards the end of a trial, minimum forward velocity was calculated for the turning episode of close turns only. For this, we used the bootstrapped rotational velocities from all corresponding trials per animal, and set a ‘turn threshold’ to the mean plus 3 times the standard deviation of the rotational velocity during episode A, i.e. before the landmark displacement. The window for calculating minimum forward velocity started at the time of landmark displacement and lasted until the rotational velocity finally fell below the ‘turn threshold’ (see also Fig. 5C).

For the segmentation of trials into curved and straight parts, trajectories of the two thorax features T1 and T3 were strongly smoothed, using a Butterworth filter (4th order, cutoff frequency: 0.25 Hz), and rotational velocities were recalculated using the filtered T1 and T3 positions. We then used the free library *Ruptures* from python (Truong et al., 2020) to segment parts of trajectories based on their rotational velocities. To this end, we chose a non-parametric rank model which was robust against outliers. The minimum segment size was set to 150 frames (3 s) and was controlled by a penalty parameter which was set to 50. Out of 4940 segments, 389 (7.9%) were shorter than 150 frames but all segments were larger than 100 frames. Performance of the splitting process was evaluated by skimming through randomly chosen trials and visually assessing whether the splitting algorithm performed as intended by looking at T1 trajectories (see Fig. S2).

Each trajectory split was resampled to contain 400 points, using linear interpolation between frames. The curvature of a given split was calculated using the metathorax marker T3 and Eqn 1:

(1) $$C=\frac{dx\cdot d^{2}y-dy\cdot d^{2}x}{{\left(dx^{2}+dy^{2}\right)}^{3/2}},$$

where d*x* and d*y* are the first derivatives, while d^2^*y* and d^2^*x* are the second derivatives of *x* and *y*, respectively. Afterwards, the radius of curvature of a segment (ROC) was calculated as 1/median(*C*).

To compare the forward velocity of similarly curved segments with and without visual guidance, we used the *curve_fit* function from the SciPy *Optimize* package (Virtanen et al., 2020) to fit the saturation curve:

(2) $$V(\mathrm{ROC})=\;\frac{V_{\max}\times \mathrm{ROC}}{K_{M}+\mathrm{ROC}},$$

where *V*_max_ is the maximum forward velocity and *K*_M_ is the ROC at half *V*_max_. This was done separately for each animal. To do so, right and left turns were pooled. The prior estimates for *V*_max_ and *K*_M_ were 40 and 100, respectively. We used Wilcoxon's signed rank test for matched samples to compare *V*_max_ and *K*_M_ between the all-white and landmark conditions.

### Step cycle detection

We trained six random forest classifiers (scikit-learn; Pedregosa et al., 2011), one for each leg, to determine whether or not a leg had ground contact. We used 1353 features to train each classifier. The feature vector contained the angles between each leg segment (using their 2D projections), the velocity of three tracked leg features (femur–tibia joint, tibia–tarsus joint and the tarsal tip) as well as the length of three leg segment projections (femur, tibia and tarsus) for all six legs (for details, see Fig. S3). In addition, we used the instantaneous body rotation, and forward and translational velocity as features. All features connected to the legs were included as time series data from 25 frames. For each frame, the feature vector therefore contained information about leg posture and velocity in 12 preceding and 12 subsequent frames. The training set to train the classifiers contained 14,916 manually labelled frames, taken from two random trials per animal. From each trial, two random 1 s sequences were used. For each frame, the state of each leg was noted as 1 if it had ‘no ground contact’, e.g. during swing, or as 0 if it had ‘ground contact’. The evaluation of the six classifiers showed only minor differences in accuracy (true positives and true negatives) between the three leg pairs (Table 1). Overall, the accuracy of all classifiers was above 95% for front legs and above 97% for middle and hind legs. For the decision ‘no ground contact’, each classifier was more likely to generate false negatives (e.g. stance instead of swing) than false positives (e.g. swing instead of stance), suggesting that leg postures without ground contact were harder to detect as such than leg postures with ground contact.

**Table 1.** Evaluation results of the six individual random forest classifiers

Confusion matrix values for ‘no ground contact’	R1	L1	R2	L2	R3	L3
True positives	24.08	24.88	18.44	18.94	18.77	18.17
True negatives	72.57	70.85	78.91	78.37	78.58	79.35
False positives	1.58	1.45	0.81	0.81	1.31	0.74
False negatives	1.78	2.79	1.85	1.88	1.34	1.75
MCC	0.91	0.89	0.92	0.92	0.92	0.92

Confusion matrix and Matthews Correlation Coefficient (MCC) values are given for each classifier. R, right; L, left; 1–3 indicate front, middle and hind legs.

The classification results were used to separate swing and stance phases. We defined the position of the tibia–tarsus joint in the last frame with ground contact as the posterior extreme position (PEP) and the first frame with ground contact after touch down as the anterior extreme position (AEP). All step parameters used in further analysis were calculated using these PEPs and AEPs. Legs were categorised as ‘inner’ and ‘outer’ legs depending on the sign of the radius of curvature of a given trajectory split (for details see ‘Data analysis at step level’, below). Left curves and corresponding step data were flipped and pooled together with right curves for all subsequent analysis.

### Step parameters and step-type classification

Throughout this paper, we will refer to a ‘step’ as the sequence beginning with an episode of ‘no ground contact’ (e.g. swing) followed by a subsequent episode of ‘ground contact’. We describe either component by means of their length (in mm), duration (in s) and direction (in deg), and implicitly assume that, on average, the parameters length and direction of the episode with ‘no ground contact’ are opposite to those of the episode ‘with contact’. Generally, we focus on the episode with ‘no ground contact’, e.g. swing phase. Note that a leg movement with ‘no ground contact’ could have different functions from a typical swing or return stroke. For instance, it could involve brief searching episodes. The ‘swing length’ and ‘swing duration’ of a step cycle were calculated as the distance and duration from PEP to AEP, respectively (Fig. 3A). ‘Swing direction’ was the direction of the vector between PEP and AEP (Fig. 3C). As a measure of how far and in which direction a leg was moved during a swing phase, we calculated the ‘swing angle’, which we defined as the angular difference between the vectors from a thorax marker to the PEP and AEP of the respective leg (Fig. 3A). Compared with the swing direction, the swing angle does not have a cyclic nature as it can take on values only between ±90 deg during planar walking.

**Fig. 3.**
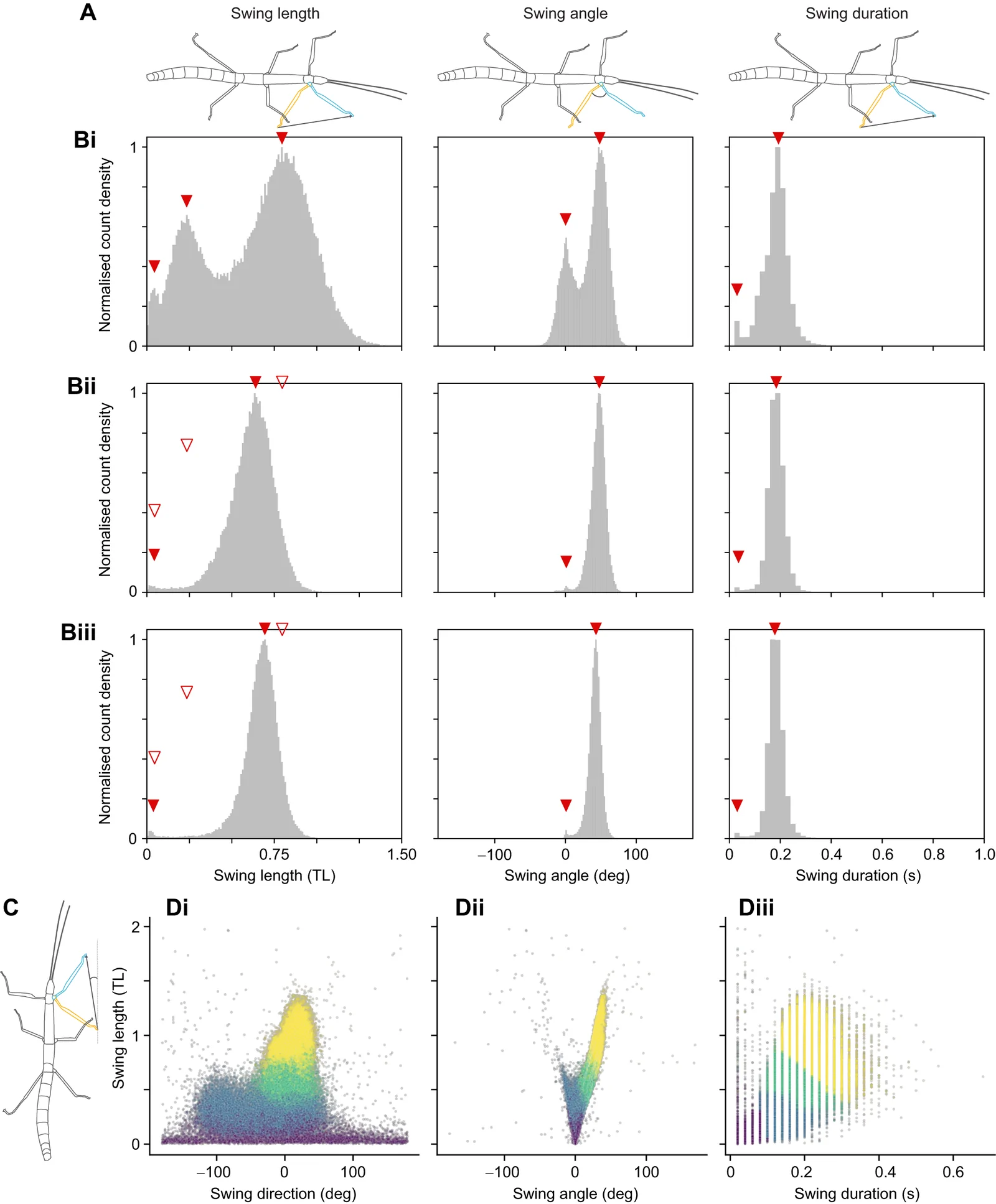
**Data-driven K-Means clustering of step classes.** (A) Schematic illustrations of step parameters used for clustering: swing length as a factor of thorax length (TL); swing angle in degrees; swing duration in seconds. The blue leg is at the anterior extreme position (AEP) and the orange leg is at the posterior extreme position (PEP). (B) Normalised count density distributions of the three parameters (illustrated in A), as used for clustering, for the front legs (i), middle legs (ii) and hindlegs (iii). Red filled arrowheads point to local maxima of the respective distribution, red open arrowheads point to local maxima of the front leg distribution. (C) Swing direction. This parameter was not used in the K-Means algorithm but to evaluate whether step classes were sufficiently distinct and showed similar properties to those described by Theunissen and Dürr (2013). The blue leg is at the AEP and the orange leg at the PEP. (D) K-Means results for swing length against swing direction (i), swing angle (ii) and swing duration (iii). Cluster 1 (purple) is labelled as ‘short steps’, cluster 2 (blue) as ‘middle steps’, and the union set of clusters 3 (green) and 4 (yellow), as ‘long steps’, yielding three step classes for subsequent analysis.

Swing length distributions of front legs were clearly non-unimodal, as observed previously by Theunissen and Dürr (2013) (Fig. 3Bi), but this non-unimodality was less clear for middle or hind legs (Fig. 3Bii,iii). Other than Theunissen and Dürr's (2013) distinction of ‘short steps’ and ‘long steps’ on the grounds of the local minimum between the two modes of the swing length distribution, we opted for a data-driven method of step class differentiation. To this end, we used the K-Means clustering algorithm from scikit-learn to classify steps according to their swing length, swing angle and swing duration. Data from all legs were scaled using the *RobustScaler* function from scikit-learn. Other than in Theunissen and Dürr (2013), no probability distribution functions were fitted to the empirical distributions; neither was the classification done separately for each leg type. Instead, we trained a single classifier for step-type classification irrespective of leg type. Finally, other than earlier step-type classification, our method involved three step parameters (length, angle and duration) instead of only one (length). As the mean swing length was smaller for middle and hind legs than for front legs, we needed to account for a potentially separate ‘long step’ cluster for these leg types, as opposed to that for front legs. For this, we included the mean of the combined distribution of middle and hind legs as a separate prior. Accordingly, the algorithm was initialised with four sets of weights, which corresponded to the four peaks of overall swing parameter distributions shown in Fig. 3Bi–iii.

The four resulting clusters differed strongly with regard to the three parameters (Fig. 3Di–iii). Cluster 1 (purple) had the shortest swing length and duration but varied strongly in swing direction: swing direction was distributed uniformly without an evident preferred direction. This was different from the other clusters, but similar to the properties of short steps as described by Theunissen and Dürr (2013). Therefore, we classified steps within this cluster as ‘short steps’. Clusters 3 (green) and 4 (yellow) did not vary much except in their mean swing length and swing angle. Moreover, both showed similar swing directions to the long steps reported by Theunissen and Dürr (2013). As the absolute size of the swing angle is largely dependent on swing length, and the two different clusters were strongly associated with distinct leg types (cluster 3 was more likely for middle and hind legs, whereas cluster 4 was more likely for front legs; see Fig. S3), we decided that these two clusters were not sufficiently distinct to be considered separate step classes. Accordingly, clusters 3 and 4 were merged into a single step class termed ‘long steps’. The steps with a medium swing length (cluster 2, blue) had a considerably different swing direction range and swing angle sign compared with steps with longer swing lengths but were also different to the short step class. Therefore, we created a third step class which we, from now on, refer to as ‘middle steps’.

### Data analysis at the step level

All subsequent analysis was done for front legs only, but see Fig. S4 for a comparison with middle and hind legs. After classifying all steps into three classes, we quantified their occurrence and potential changes in swing direction during straight walking as opposed to turning. To this end, we separated curved from straight trajectory segments according to their ROC.

To compare whether the three step types occurred at different frequencies for inner and outer legs (with regard to the curvature), and whether this changed as a function of curvature, we introduced an asymmetry measure as the dot product *A*=(*S*_inner_, *M*_inner_, *L*_inner_)·(*S*_outer_, *M*_outer_, *L*_outer_), where *S*, *M* and *L* are the percentages of short, middle and long steps, respectively. Percentages were calculated as means per animal for the two 3 s episodes, A and B (Fig. 1).

To address the question of whether leg movements were adapted as a function of total turn angle or rather of maximum curvature, we compared turning responses to 60 deg or 120 deg displacements in landmark position. For this, we calculated the change in swing direction as well as the change in its variance, using the circular summary statistics ‘mean swing direction’ and ‘length of the resultant mean direction vector’ for a trajectory segment, again as means per animal and for both episodes A and B. To compare the latter, we calculated the mean differences of the circular summary statistics.

To compare the frequency of short steps during spontaneous and visually guided turns, and to relate this to short step frequency in earlier studies, we calculated the fraction of short steps for the trajectory segment of a given range of ROC (see ‘Data analysis at the trajectory level’, above). Again, this was done as means per animal per bin. For statistical comparison between trials with and without a landmark, i.e. visually guided as opposed to spontaneous turns, we used a Friedman test across all bins to test the null hypothesis that the two conditions were equal. This was followed by pair-wise *post hoc* Wilcoxon signed-rank tests for comparisons within bins.

## RESULTS

### Path length, curvature and forward velocity differ among conditions

Animals readily and reliably approached the visual landmark, i.e. the black bar, if present (Figs 1 and 4; Fig. S1). The stepwise change in landmark position did not decrease the likelihood of an animal approaching the wall or landmark (Table S1). However, for the larger displacement, the average likelihood of approaching the target decreased by 20% compared with that for the smaller displacement (Fig. 4B; Table S1). In the all-white condition, animals approached the arena wall less often compared with trials in which a landmark was present (Fig. 4B; Table S1). Without visual guidance, animals tended to walk around in circles (Figs 2A and 4A). Trajectories in the all-white condition sometimes comprised one or multiple 360 deg turns (for example, see Fig. 1C). Overall, the probability of an animal being ‘on target’ or ‘at wall’ seemed to be unaffected by both the starting position of the animal in the arena and the direction of the landmark displacement (Fig. 4B,C; Table S1). Therefore, we decided to pool both the starting positions and the displacement directions for the remaining analysis. Animals did not show a persistent orientation towards the original landmark position in trials with a change in landmark position, but re-oriented towards the new target instead (Fig. 4A,B). For all-white trials, we found no support for a preferred final position as in landmark trials.

**Fig. 4.**
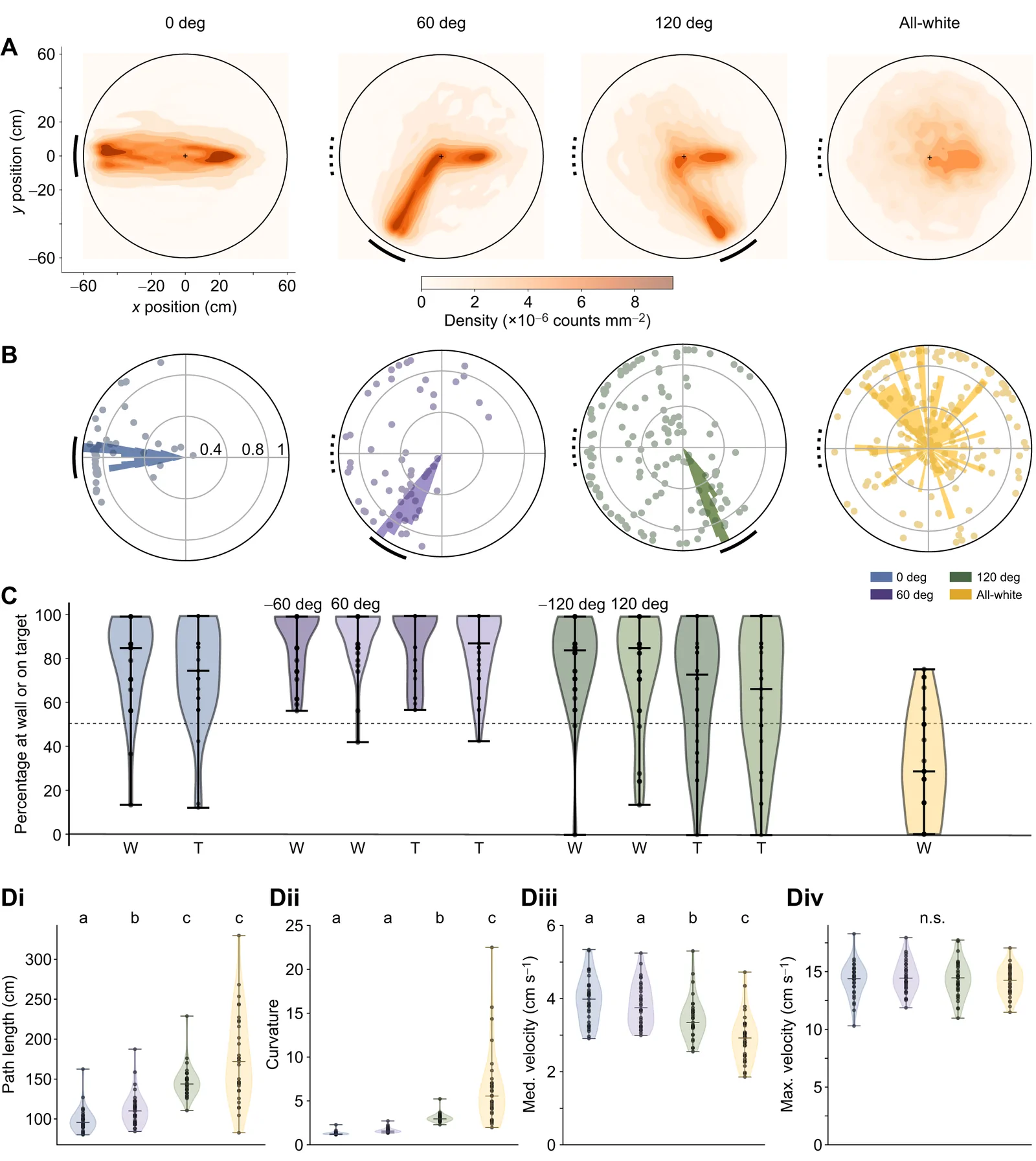
**Final head position and likelihood of approach depend on landmark presence.** (A) Kernel density estimation plots across all trials. Solid black lines outside the circle show the width and position of the black bar presented; dotted black lines show the first position of the black bar before it was displaced in 60 deg and 120 deg trials. In the all-white trial, the dotted line indicates the start orientation of the animal, as no black bar was present at any time. (B) Rose plots showing the final head position of all trials that terminated at the arena wall (‘at-wall’ trials). Coloured circles show the end positions of trials in which animals were not ‘at wall’. Labelled circular lines indicate fractions of the arena radius. (C) Violin plots showing the likelihood of an animal being ‘at wall’ (W) and ‘on target’ (T). Note that ‘on-target’ trials are a subset of ‘at-wall’ trials. Each data point represents one mean per animal (*N*=31). ±60 deg and ±120 deg trials are shown as separate violin plots but were pooled for the rose plots in B. The detailed statistical results can be found in Table S1. (D) Violin plots showing the mean across on-target trials of 30 individual animals for three different measures for path length (i), curvature (ii), median forward velocity (iii) and maximum forward velocity (iv). n.s. indicates non-significant Friedman test for the median in repeated samples. In case of a significant Friedman test, letters a to c label samples with equal median according to *post hoc* Wilcoxon tests for matched pairs. Each data point represents one mean per animal. Statistical results as well as mean and s.e.m. can be found in Table S2.

Generally, the overall curvature of a trial was increased for all-white trials compared with trials with a landmark present (Fig. 4Dii; Table S2). The 60 deg condition did not differ from the 0 deg condition regarding median forward velocity, maximum forward velocity or overall curvature present (Fig. 4D; Table S2). In contrast, the 120 deg and all-white conditions both showed an increased curvature and a decreased median forward velocity (Fig. 4Dii,iii; Table S2). The maximum forward velocity was not affected by a change in landmark position or the absence of a landmark (Fig. 4Div; Table S2).

### Displacement of landmark affects turning duration more than rotational velocity

To address the question of how turning velocity and duration scale with the amplitude of a turn, we compared three measurements: the maximum rotational velocity and its timing, as well as the duration of the turn. As forward velocity has been reported to decrease or increase during turning, depending on the type of turn (e.g. Yang et al., 2024), we analysed whether rotational and forward velocities followed a similar time course and whether forward velocity also scaled with the amplitude of a turn. Overall, we found that trials in which the landmark was moved by 120 deg appeared more variable in the approach to the new landmark position. This was particularly visible in the spatial distribution of episode B, which covered the largest rotation throughout a trial (Figs 2A and 5Aii). We separated trials in which animals turned away from the target and approached it coming from the other side. These ‘away’ responses were more abundant in 120 deg trials (Fig. 5B). To further constrain the comparison to similar response types, we distinguished three categories according to where the maximal rotational velocity occurred: close to, intermediate (middle) and far from the midline of the arena. Again, the number of trials falling into these three categories varied between 60 deg and 120 deg turns. While animals turned close to the midline in 90% of the 60 deg trials, they did so in only 46% of 120 deg trials. The time courses of rotational velocities varied considerably for the three different categories for 120 deg trials. Close turns showed a fast increase in rotational velocity followed by a short plateau at maximum rotational velocity and a fast but gradual decrease. In middle turns, animals rotated throughout the whole trial. In far turns, the onset of rotation was delayed by approximately 3 s (Fig. 5C, right).

**Fig. 5.**
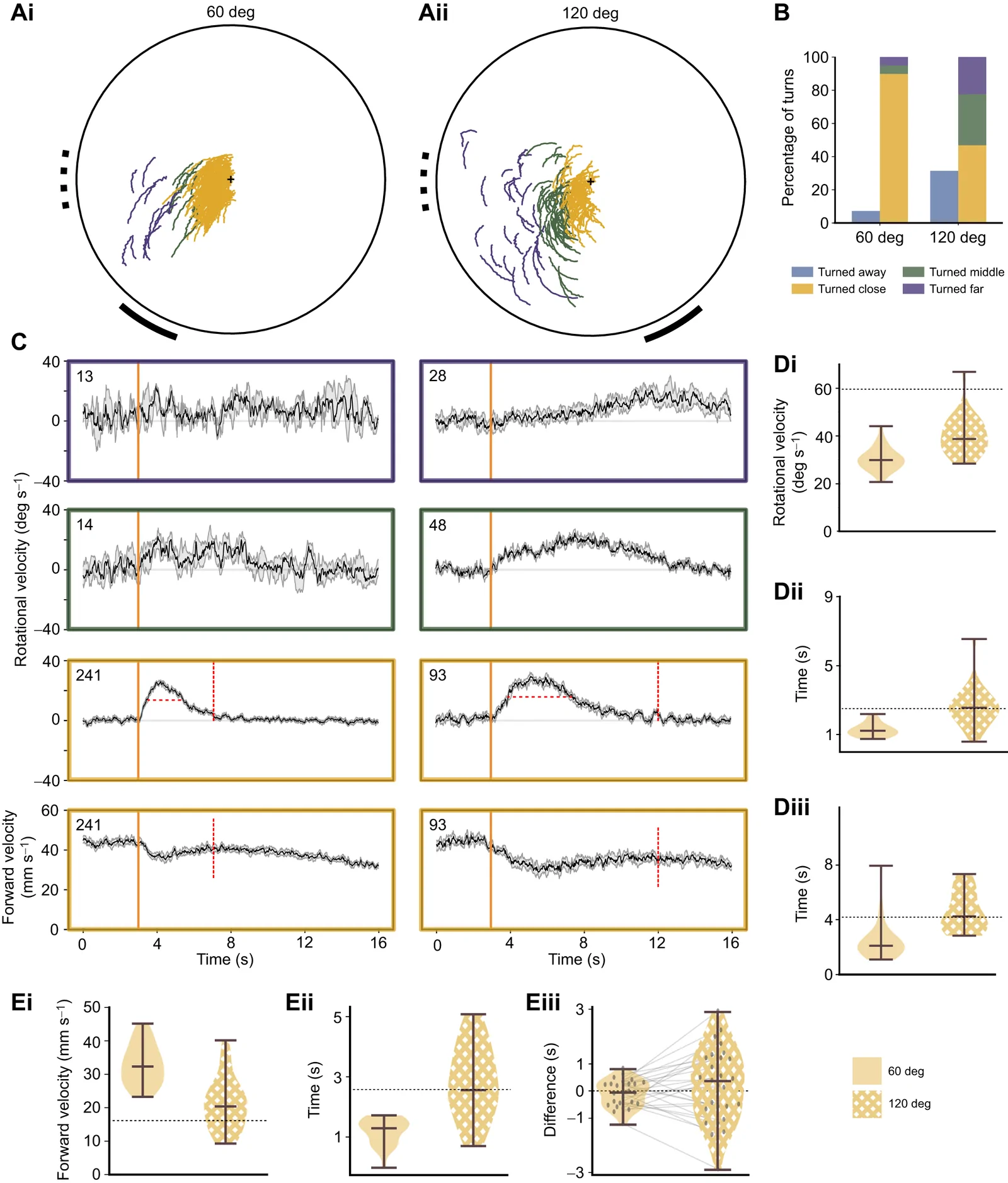
**Maximum rotational velocity is larger and occurs later with a larger landmark displacement.** (A) Top view of circular arena with *xy*-positions of the prothorax marker during episode B. All ‘central’ trials with turns towards the displaced landmark are shown. Solid black lines outside the circle show the width and position of the landmark presented; dotted black lines show the initial position of the landmark before it was displaced. The plot only includes ‘on-target’ trials. The black cross indicates the midpoint of the arena. Trajectories are colour coded according to the location of maximal turning. (B) Bar plots showing the percentage of ‘central’ trials in which an animal turned away from the new landmark position (blue), turned close to the midpoint (yellow), turned in an intermediate (middle) range (green) or turned far away from the midpoint (purple). (C) Bootstrapped rotational velocity time courses. Black lines show the mean and grey lines show the 95% confidence interval around the bootstrapped mean. Numbers in the upper left corner indicate the number of trials used for bootstrapping. Coloured boxes around the plots correspond to the category of their turn location, as in B. For ‘close’ trials (yellow), the second plot also shows the forward velocity time course. Horizontal red dashed lines schematically show the ‘full width at half maximum’. Vertical red dashed lines show the time interval used for the calculation of minimum forward velocity. (D) Violin plots showing the maximum rotational velocity (i), the timing of maximum rotational velocity after displacement of the landmark (ii) and the width at half maximum as a measure of rotation duration (iii). To assess parameter scaling with turn angle, dotted lines indicate twice the median value of 60 deg turns. (E) Violin plots showing the minimum forward velocity (i), the timing of minimum forward velocity after displacement of the landmark (ii) and the temporal difference between the minimum forward velocity and maximum rotational velocity (iii). The grey dotted line indicates the zero-time difference. Grey dots show individual data points and grey solid lines connect data points of the same animals in both conditions. Values used for statistical comparison were means per animal. *N*=29.

The quantitative comparison of 60 deg and 120 deg turns was confined to close turns. To distinguish between the alternative hypotheses that turns with twice the turn angle involved either 2-fold rotational velocity (Fig. 5Di) or double the duration, we compared the mean parameters per animal against the theoretical predictions for peak rotational velocity (Fig. 5Di), time of peak velocity (Fig. 5Dii) and the turn duration at more than half the peak velocity (full width at half maximum; Fig. 5Diii). On average, animals turned for nearly twice as long in 120 deg turns compared with 60 deg turns (*P*<0.0001, mean±s.e.m.: 60 deg: 2.44±0.26 s, 120 deg: 4.57±0.27 s; Fig. 5Diii), suggesting that animals covered a larger turn angle by increasing turn duration rather than speed. However, maximum rotational velocity was increased too for larger displacement magnitudes, albeit with mean velocities well below the predicted double velocity (*P*<0.0001, mean±s.e.m.: 60 deg: 30.35±0.91 deg s^−1^, 120 deg: 40.26±1.54 deg s^−1^; Fig. 5Di). In addition, the maximum rotational velocity was reached later in 120 deg trials (*P*<0.0001, mean±s.e.m.: 60 deg: 4.28±0.07 s, 120 deg: 5.57±0.21 s; Fig. 5Dii). We conclude that our results support the hypothesis that larger turn angles are covered by proportionally longer turns (here: a factor of 1.87 compared with an expected 2.0), but that the scaling of turn parameters is non-linear. This non-linearity results in increased mean rotational velocity by a factor of 1.32.

For both turn angles, forward velocity decreased alongside the increase in rotational velocity (Fig. 5C). Here, minimum forward velocity also scaled non-linearly with turn angle as forward velocity was decreased more for larger turn angles by a factor of 1.54 (*P*<0.0001, mean±s.e.m.: 60: 32.98±1.18 mm s^−1^, 120: 21.35±1.49 mm s^−1^; Fig. 5Ei). Minimum forward and maximum rotational velocity shared a similar timing; however, they did not occur at exactly the same time (see individual data points in Fig. 5Eiii). The difference in timing of minimum forward velocity and maximum rotational velocity did not differ from zero for either displacement magnitude (*P*_60_=0.34, *P*_120_=0.51, mean±s.e.m.: 60 deg: −0.11±0.09 s, 120 deg: 0.2±0.26 s; Fig. 5Eiii). However, as the distribution of the difference in timing was nearly symmetrical around zero, albeit never being zero, the minimum forward velocity was either followed or preceded by the maximum rotational velocity. As before, 120 deg turns were more variable than 60 deg turns (Levene's test: *P*<0.0001; Fig. 5Eiii). Especially for 60 deg turns, the timing of these two events was less than a second apart for the majority of the animals, indicating a linkage between rotational and forward velocity, albeit with two different relationships: Maximal rotational velocity leads minimum forward velocity or vice versa. These ‘strategies’ were not consistent for individual animals (Wilcoxon signed-rank test: *P*=0.0005, indicated by zero crossings of grey lines in Fig. 5Eiii), revealing that animals changed strategies between different trials. In summary, we found that both rotational and forward velocity scale with larger turn angles in a non-linear manner and that the two parameters share a similar time course. However, there was no evidence for a strict coupling of forward and rotational velocity during turning.

### Presence of a visual landmark increases velocity and curvature

Our second objective was to test whether spontaneous turns differ from visually guided turns. Like guided turns, spontaneous turns varied in curvature. Accordingly, we addressed the question of whether visual guidance in the form of a landmark is associated with an increased forward velocity for similar curvatures. To this end, we divided each trial into segments of different curvature and compared forward velocities between episodes of equal curvature with or without visual guidance.

First, visual guidance in the form of a black landmark increased the number of straight segments within a trial (Fig. 6). While 83.4% of segments in all-white trials (Fig. 6B) were within a ROC of ±600 mm, only 58.1% of guided trials (Fig. 6A) fell into that range (for visualisation of ROC, see Fig. S2). As in previous results, forward velocity varied by a large amount among animals (Figs 6C and 3Diii) and was linked to curvature (Fig. 5Ei).

**Fig. 6.**
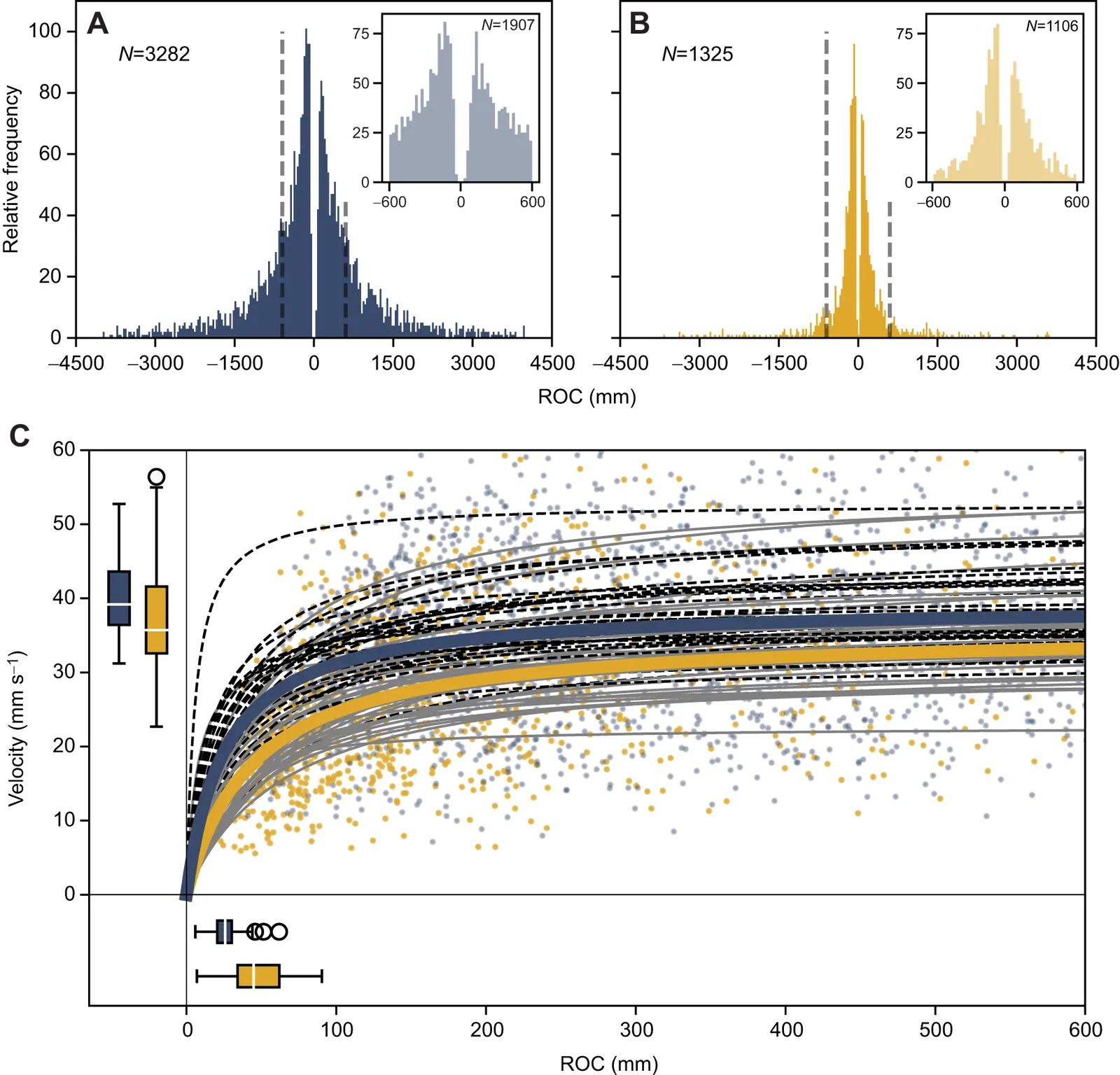
**Guided turns are faster than spontaneous turns with the same radius of curvature.** (A,B) Histograms showing the number of trajectory segments with a given radius of curvature (ROC) for guided (A, blue) and spontaneous (B, yellow) turns. Insets show relative frequencies of the area marked by the vertical dashed grey lines on an expanded scale. Here, 55% of all segments were clockwise turns (right) and the remaining 45% were counter-clockwise turns (left). Bin width=30 mm; *N*=30 animals. (C) Estimated saturation functions per animal are shown by grey solid lines (spontaneous condition) and black dashed lines (guided conditions). Thick blue and yellow lines show pooled saturation functions using the median maximum forward speed (*V*_max_) and the ROC at half *V*_max_ (*K*_M_) for guided and spontaneous conditions, respectively. Blue and yellow dots in the background show values of all trajectory segments clipped to ROC <600 mm and *V*_max_ <60 mm s^−1^. Box plots show distributions of fitted parameters *V*_max_ and ROC, with boxes showing IQR and whiskers showing minimum and maximum after exclusion of outliers. Open circles show outliers located more than 1.5×IQR beyond the box. Note that saturation curves were fitted to non-clipped data ranges.

To quantify the curvature–velocity dependence, we fitted separate saturation functions for each animal and statistically compared the maximum forward speed (*V*_max_) and the ROC at half *V*_max_ (*K*_M_). We found that both, *K*_M_ and *V*_max_ differed between the spontaneous and guided condition (*P_V_*_max_=0.03, *P*_K_M__<0.0001). Without visual guidance, the average maximum forward velocity was decreased (*V*_max_ guided: 40.27±1.01 mm s^−1^, *V*_max_ spontaneous: 38.05±1.43 mm s^−1^; means±s.e.m.) and the ROC at half *V*_max_ was increased (*K*_M_ guided: 27.33±2.25 mm, *K*_M_ spontaneous: 47.99±3.86 mm; means±s.e.m.), indicating that paths of similar curvature were walked at a higher forward velocity if a landmark was present (Fig. 6B).

### Stick insects use three classes of steps during unrestrained locomotion

To address our questions at the level of single steps, we first had to detect and classify all steps. Although this was done for all six legs, our hypotheses focused on the front legs in particular, such that we will report results on the front legs only. All material concerning the middle and hind legs can be found in Figs S3 and S4.

Generally, we found strong variation of step length during unrestrained turning on a planar surface. Associated step length distributions were not unimodal, suggesting the presence of distinct step classes. However, step length distributions differed from those found in studies that used ‘straight walkway’ setups (Theunissen and Dürr, 2013; Theunissen et al., 2015; Bläsing and Cruse, 2004b). Instead of a bimodal distribution with short and long steps, we found a trimodal distribution of swing length (Fig. 3Bi). To check whether the medium swing length mode was related to turning rather than to straight walking, we first separated steps from nearly ‘straight’ segments, i.e. with a ROC larger than 600 mm, from those of ‘curved’ segments with a smaller ROC. As steps of medium length occurred in straight and curved segments alike, though with different frequencies (Fig. 7A,C), we concluded that a binary distinction between long and short steps was inappropriate for our data. Following data-driven categorisation based on the three step parameters length, duration and angle (see ‘Step parameters and step-type classification’, above; Fig. 3), we found that steps could be divided into three step classes that corresponded to the three modes of the step length distribution: short, middle and long steps.

**Fig. 7.**
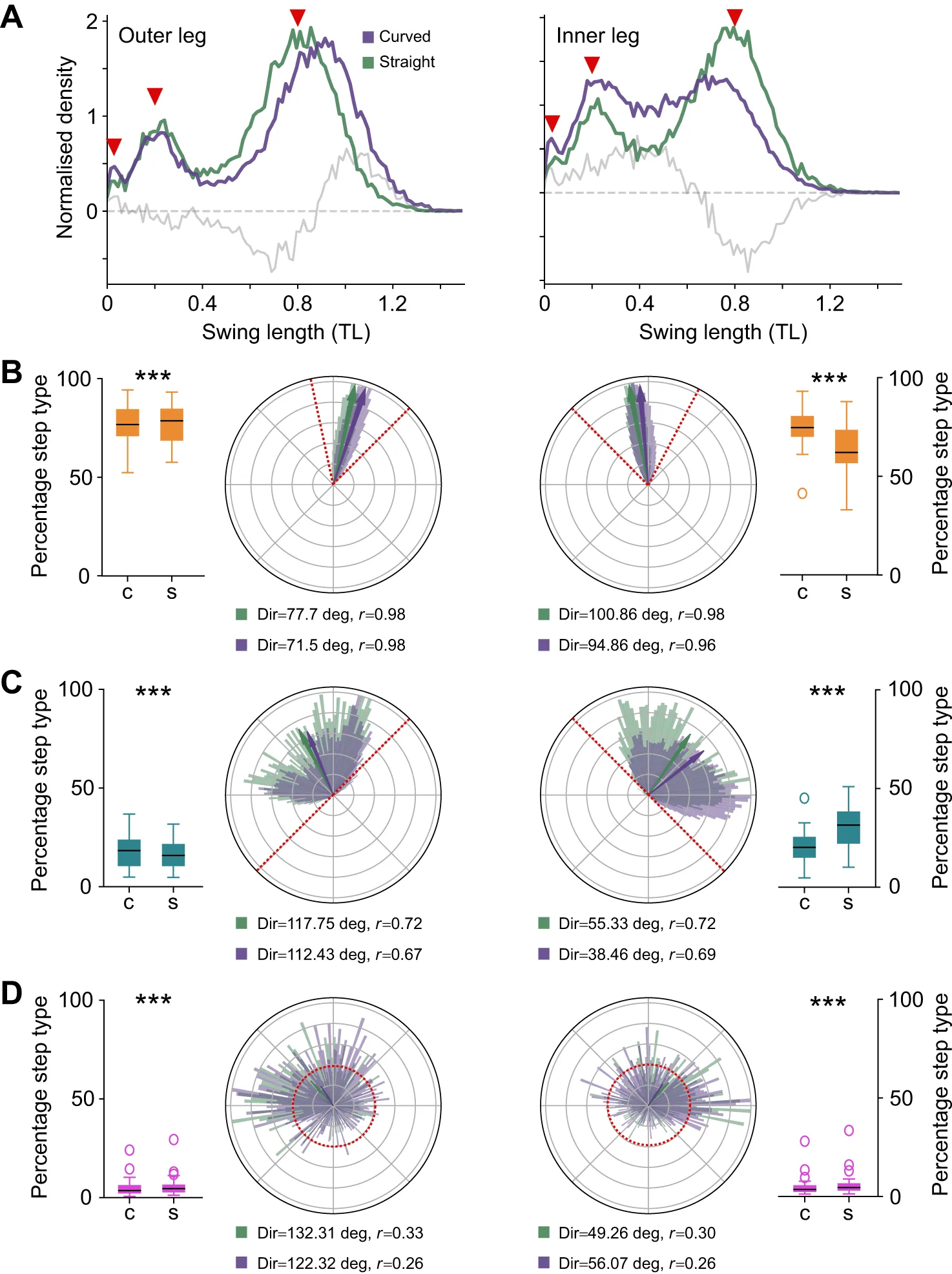
**Stick insects use three classes of steps during unrestrained planar walking.** (A) Density plots showing the distribution of swing lengths of front legs (left: outer leg, right: inner leg) in curved (purple, ROC ≤600 mm, *N*=32, *n*_steps_inside_=25,202, *n*_steps_outside_=21,896) and straight segments (turquoise, ROC >600 mm, *N*=32, *n*_steps_inside_=16,375, *n*_steps_outside_=15,951). Red arrows indicate the local maxima of the combined front leg distributions, as in Fig. 3Bi. The grey solid line shows the difference between the curved and straight conditions. The grey dashed line marks zero density or difference. *N*=32. (B–D) Circular histograms showing the swing direction of front leg steps (left: outer leg, right: inner leg) in curved (purple) and straight (green) segments for long steps (B), middle steps (C) and short steps (D). The mean direction (dir) and the concentration around the mean direction (*r*) are given below each histogram. Boxplots show the percentage of a step type for straight (s) and curved (c) segments, with boxes showing IQR and whiskers showing minimum and maximum; *N*=31 per-animal means. Asterisks indicate *P*<0.001 of a Wilcoxon test for matched pairs. For statistical results, see Table S3.

When comparing the properties of these three step classes, swing directions of short and long steps were similar to findings of previous studies: while long steps were directed towards the front with a slight medial slant (Fig. 7B), short steps had little to no directional bias (Fig. 7D). Other than that, middle steps were directed laterally and, similar to long steps, slightly medially. This was the case for both inner and outer legs. However, unlike short steps, they never pointed into the medio-caudal half of the unit circle. The variance in swing direction was larger for middle steps than for long steps, but smaller than that for short steps (as reflected by mean vector length *r* in Fig. 7B–D). During curve walking, the mean swing direction of long steps was shifted towards the direction of the curve for both inner and outer legs. In middle steps, this shift in mean direction was stronger for the inner leg. Here, the frequency of middle steps also increased during curve walking, while the percentage of long steps decreased accordingly (see boxplots in Fig. 7B–D). The frequency of all three step classes changed during curve walking (Table S3).

Additionally, we found that left–right asymmetry of long and middle step frequencies was prominent during curve walking, where inner legs take fewer long steps and more middle steps compared with outer legs (long steps: −1.61, middle steps: 1.84, Cohen's *d*). We found a similar relationship for near-‘straight’ walks, although with low effect sizes (long steps: −0.27, middle steps: 0.31, Cohen's *d*) as this effect is probably linked to the fact that ROCs of 600 mm are still shallow curves. Taken together, we found that step-type frequency for all legs changed from straight to curve walking, as well as an asymmetry in step-type frequency between inner and outer legs during curve walking.

### Swing direction is altered more strongly for larger turn angles

As the previous result indicated that swing direction as well as step-type frequency change during curve walking, we wanted to find out how the scaling of turning responses is carried out at the level of individual steps. Accordingly, we calculated the change in swing direction as well as the change in variance of swing direction from the ‘straight walking’ episode A to the ‘curve walking’ episode B, as distinguished in Figs 1 and 2. To relate this to the body kinematics of Fig. 5, we only used ‘central’ trials, which were categorised as ‘close’ turns ‘towards’ the landmark.

For both conditions with target displacement, turn angles were the same during ‘straight’ episode A, and significantly increased during ‘curved’ episode B (Fig. 8A). Throughout the 3 s of ‘curved’ episode B, animals covered an approximately 50% larger turn angle in 120 deg trials compared with 60 deg trials (Fig. 8A). The different turn angle between straight and curved episodes was accompanied by a corresponding change in left–right asymmetry of step-type frequency, as reflected by the smaller dot product of the frequency vectors for inner and outer legs (Fig. 8B; see ‘Data analysis at the step level’, above). Generally, left–right asymmetry increased during turning. It was significantly larger for 120 deg trials than for 60 deg trials (Fig. 8B).

**Fig. 8.**
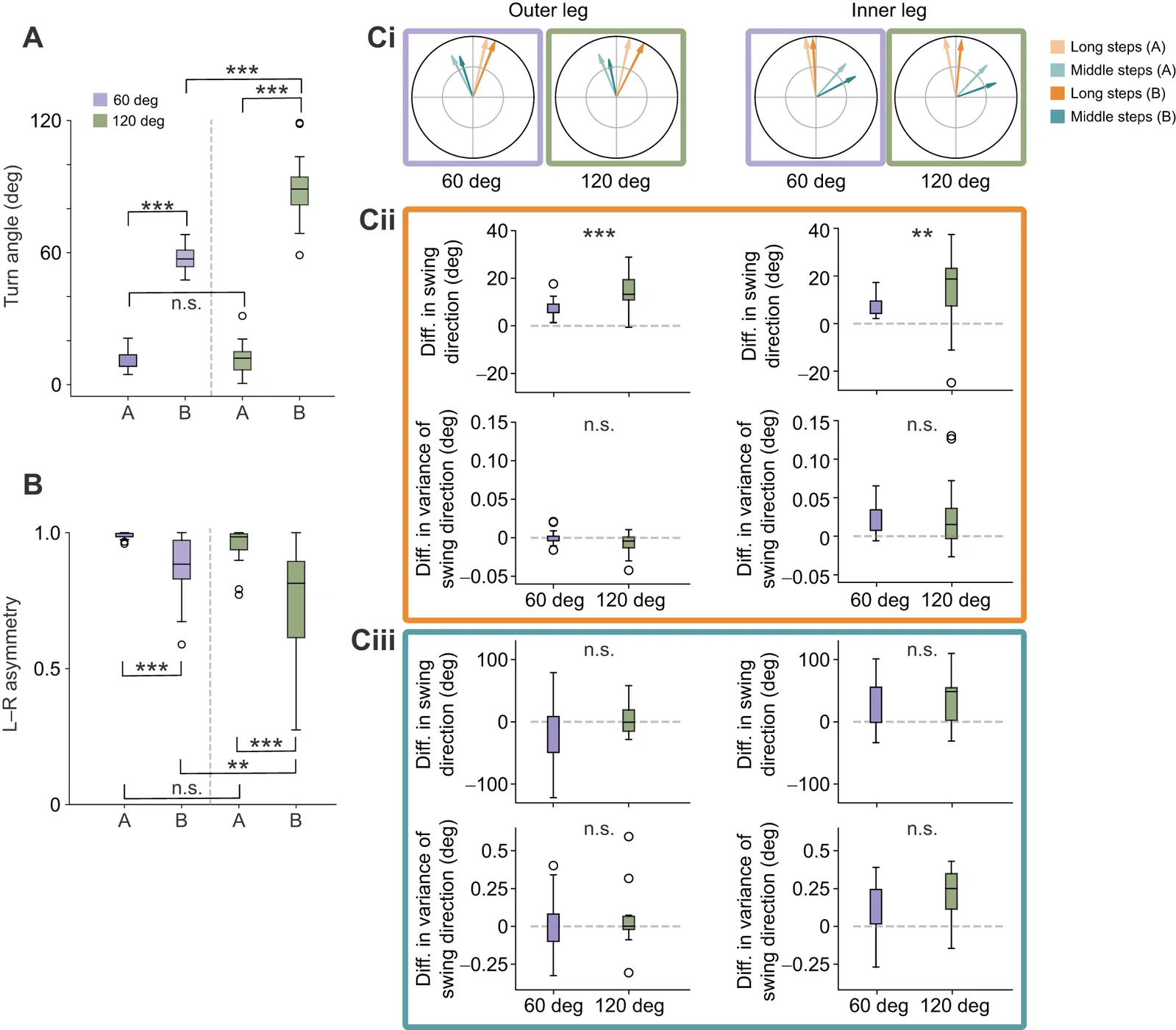
**Swing direction changes are larger during prolonged rotation.** (A) The turn angle covered during the 3 s episodes A and B differs between 60 deg (purple) and 120 deg (green) trials. (B) Left–right asymmetry between step class frequencies of inner and outer front legs during straight episode A and curved episode B differs between 60 deg (purple) and 120 deg (green) trials. (Ci) Median swing direction across all animals of two step classes (orange: long steps, turquoise: middle steps) for both front legs (left: outer leg, right: inner leg) during 60 deg (purple) and 120 deg trials (green). Arrows of lower opacity belong to the straight episode A, those of higher opacity to the curved episode B. The inner grey circle shows *r*=0.5. The outer black circle shows *r*=1. *N*=29. (Cii) Top: the difference in swing direction of long steps between episodes A and B as means per animal for outer leg (left) and inner leg (right). Bottom: the difference in variance of swing direction of long steps between episodes A and B, as means per animal (left: outer leg, right: inner leg). Each plot compares 60 deg (purple) against 120 deg trials (green). (Ciii) Same as Cii, but for middle steps. Box plots show IQR, with horizontal line showing the median, and whiskers show maximum and minimum. Conditions were compared by Wilcoxon test for matched pairs on means per animal. **P<0.01, ***P<0.001; n.s., not significant. For statistical results and all mean and s.e.m. values, see Tables S4–S6.

During turning, swing direction changed significantly more in 120 deg trials compared with 60 deg trials for both inner (Fig. 8Cii; 60 deg: 7.07±0.63 deg, 120 deg: 14.7±2.62 deg; means±s.e.m.) and outer legs (60 deg: 7.91±0.61 deg, 120 deg: 14.38±1.24 deg). Swing direction of the inner leg was directed further towards the inside of the curve, with a more than 2-fold larger angle in 120 deg trials (factor 2.08). In outer legs, the increase was also significant, though slightly smaller (factor 1.82; Fig. 8Cii). The turning-related change in variance of swing direction among long steps was larger in inner legs compared with outer legs for 60 deg trials (inner: 0.0243±0.033, outer: −0.0007±0.0014; means±s.e.m.) and for 120 deg trials (inner: 0.0226±0.0071, outer: −0.0062±0.0022; Fig. 8Cii) but similar between the two conditions.

Unlike for long steps, the turning-related change in swing direction of middle steps did not differ either between inner and outer legs or between 60 deg and 120 deg turns (Fig. 8Ciii). Although the variance of swing direction changes was very large for both turn angles, it did not differ significantly (Fig. 8Ciii).

Overall, we found that during turning, long steps of both inner and outer legs were rotated towards the direction of the turn. This effect was nearly 2-fold larger for 120 deg turns compared with 60 deg turns. At the same time, swing direction of middle steps did not change (Fig. 8C), despite a turning-related change in step-type asymmetry between inner and outer legs (Fig. 8B).

### Front legs execute more short steps during tight turns without visual guidance

Finally, to test whether short steps might be used as intermitted ‘searching steps’ in a walking scenario without visual guidance, we compared the relative frequency of short steps between trials with visually guided turns and trials with spontaneous turns, i.e. between trials with and without a visual landmark, respectively. To account for the different ROC values, we used four bins of ROC from the segment analysis in Fig. 6.

Overall, we found a small but statistically significant increase in the relative frequency of short steps in all-white trials compared with visual landmark trials (Fig. 9; Friedman test: *P*<0.0001). *Post hoc* paired tests for different ranges of ROC revealed that this significant difference was mainly carried by differences between tight turns. In strongly curved segments (ROC <200 mm), short steps were about 1.5% more frequent in all-white trials than in landmark trials (mean±s.e.m.: visually guided 4.6±0.57%, white 6.08±0.73%, *P*=0.0024). For shallower curves and straight segments, the number of short steps was not different for the two conditions (after Bonferroni correction).

**Fig. 9.**
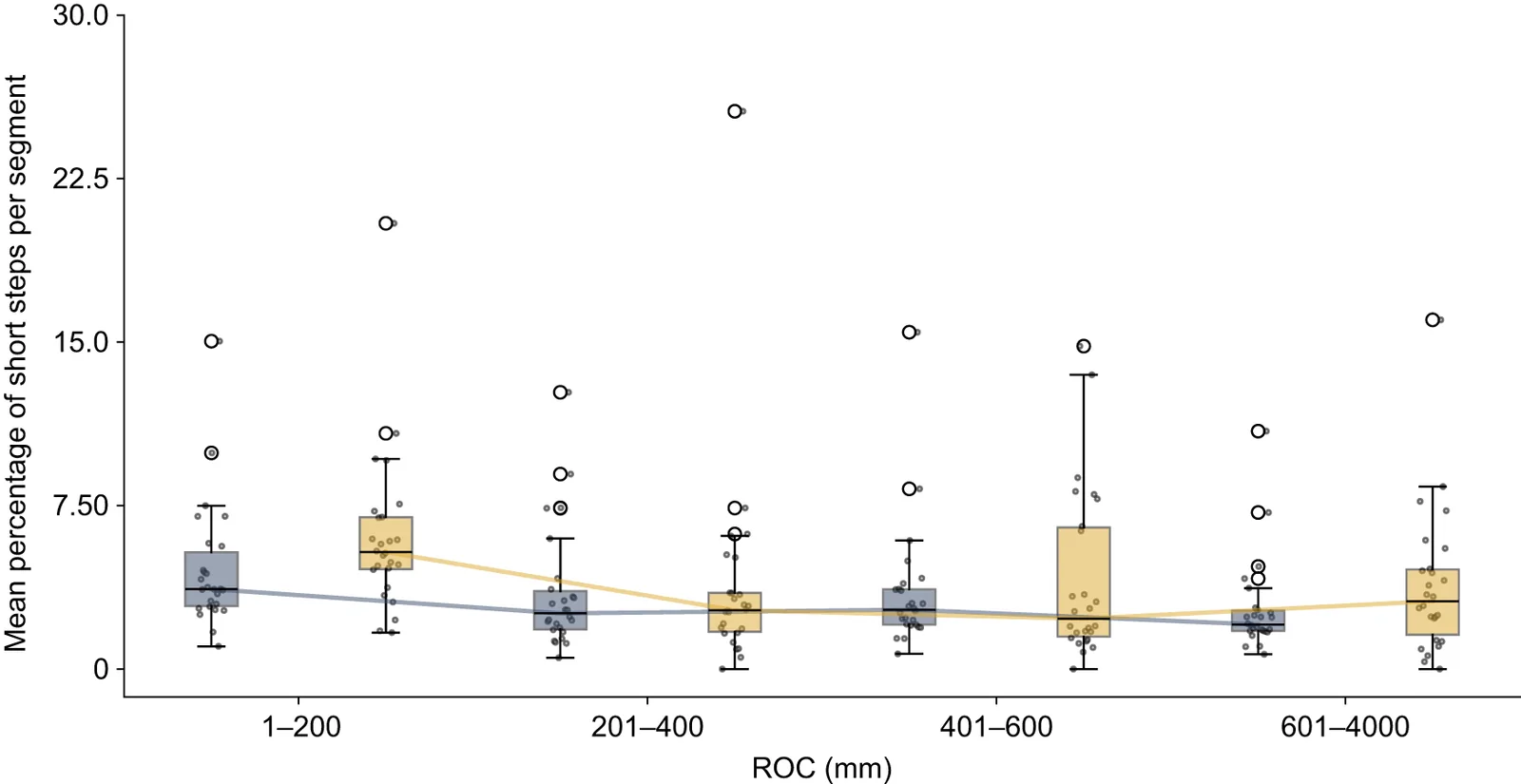
**Short steps are more frequent in spontaneous tight turns*.*** Boxplots show the percentage of short steps for path segments of four different ROC ranges in spontaneous (yellow) and visually guided (blue) curves. Segments were grouped according to their ROC as means per animal per condition; *N*=26. Boxes show IQR, with horizontal line showing the median, whiskers show minimum and, because of outliers, 1.5× the IQR above the 3rd quartile. For statistical results and all mean and s.e.m. values, see Table S7.

## DISCUSSION

Flexible walking behaviour allows animals to change their heading according to context and across a range of curvatures. Here, we investigated contextual effects in the form of visual guidance, and magnitude-related effects in the form of scaled turning responses. For both of these aspects, we linked the (high) behavioural level of body trajectories to the (low) causal level of step parameters (with a focus on front legs). To induce different turn magnitudes, we modified a landmark displacement paradigm introduced by Rosano and Webb (2007), constraining both onset time and desired turn angle of unrestrained walking stick insects. To our knowledge, this is the first study to address the scaling of turning responses towards stationary targets in unrestrained walking.

### Turning responses scale non-linearly with turn angle

As shown by our preliminary experiment (Fig. 1B), stick insects reliably turned towards displaced landmarks up to an angle of 150 deg, though larger displacement magnitudes affect the likelihood of ‘close turns’, i.e. near the arena centre (Fig. 5B). For ‘close turns’, we found that turning duration and maximum turning velocity scaled with the displacement of a landmark in a non-linear manner, as turning duration was affected to a larger extent (1.87×) than maximum turning velocity (1.32×) (Fig. 5). Similar to findings in spiders (Land, 1972), the rotational velocity was not constant between or within turns, indicating that the turn towards a stationary target is not simply scaled according to an error signal (e.g. target eccentricity).

As the scaling factor for turning duration was larger than that for rotational velocity (Fig. 5), one might expect rather small changes in leg movement parameters because turning duration should correlate with the number of steps needed to cover the larger turn angle. However, we found that changes in swing direction of both front legs were up to twice as large in the 120 deg condition compared with the 60 deg condition (Fig. 8C), suggesting that even small increases in rotational velocity arise from strongly altered leg movements. In addition, as the left–right asymmetry of step types changes significantly for larger turn angles, the mix of step parameters was altered differently for inner and outer legs, depending on turn angle. This is consistent with findings by Yang et al. (2024), who observed different combinations of ‘leg gestures’ for turn types of different curvature. Indeed, these authors could relate narrow ‘pivot’ versus shallow ‘swerve’ turns in spontaneously turning *Drosophila* to activity modulation of identified descending interneurons. Though both turn types were identified through transient increases of rotational velocity, the associated steering manoeuvres involved either an increase (for shallow turns) or a decrease (for narrow turns) in forward velocity (Yang et al., 2024). For *C. morosus*, Dürr and Ebeling (2005) found no change in forward velocity during visual motion-induced turns in tethered walking, but a small but significant increase in sideward velocity. In contrast to both of these studies, we found no evidence for an increase in forward velocity but, rather, a scaled reduction of forward velocity, depending on overall turn angle. It is possible that these differences correspond to different walking conditions (free versus tethered) and/or paradigms (spontaneous, motion-induced or target-induced, see below). As differences in the timing of minimum forward and maximum rotational velocity did not occur in a fixed sequence, there is no evidence for a strict coupling of forward and rotational velocity during turning. Rather, different animals reached their maximum rotational velocity either before or after reducing their forward velocity to a minimum (Fig. 5Eiii). Thus, different individuals show different strategies and tuning of turning parameters to walk the same curve. However, as individuals also changed strategies between 60 deg and 120 deg turns, idiosyncrasies as observed for straight walking in *Drosophila* (Godesberg et al., 2024) are unlikely to be the driver of these different strategies. Instead, these findings suggest motor flexibility during turning. Taken together, the number of parameters that change during target-induced turning and their different scaling with turn magnitude support the idea that locomotion models should combine more than one mechanism to govern turning. Moreover, these mechanisms need to be adaptive to account for continuous scaling of curvature at different walking speeds, while retaining flexibility for multiple possible combinations of parameter scaling to change heading. Our study sets the stage for a detailed sequel analysis on changes in inter-leg coordination (Dürr, 2005; Rosano and Webb, 2007; DeAngelis et al., 2019) for turns of different radii as well as on the flexibility of the transition from straight to curve walking.

### Distinct step classes during turning

Adaptive walking has been shown to involve proprioceptive feedback in addition to changes in centrally generated rhythmic motor patterns (for reviews, see Ritzmann and Büschges, 2007; Büschges et al., 2008), in correspondence with descending input from brain neuropils (Cruz and Chiappe, 2023). In turning stick insects, the strength in inter-leg coupling has been shown to change differently for distinct leg pairs, with a left–right asymmetry as well as a pronounced effect on front leg coupling (Dürr, 2005). A near-complete disruption of a six-legged gait occurs in tight on-the-spot turns, e.g. when turning on a twig (Cruse et al., 2009b). In accordance with possible gait disruption, and the special role of the front legs, our present paper focused on front legs only (but see Figs S3 and S4). This is further justified by the occurrence of distinct step types (Theunissen and Dürr, 2013; Theunissen et al., 2015; Bläsing and Cruse, 2004b), the identification of which requires knowledge of step parameter stochastics. While neglecting temporal coordination among legs, this approach has the advantage of incorporating aspects of spatial coordination as well as being applicable to walking sequences where no distinct gait is identifiable.

Although step parameters of both swing and stance phase change with corresponding magnitudes during turning (Dürr and Ebeling, 2005), a recent study showed that turns toward static visual landmarks tend to be initiated by a change in swing direction (Bigge et al., 2026). Accordingly, our step parameter analysis focused on swing phase parameters. Contrary to the studies by Theunissen and Dürr (2013) and Theunissen et al. (2015), we found a trimodal step length distribution, comprising a third, previously undetected step class (Fig. 7). If we had applied the same step length criterion to separate steps into two classes as done by Theunissen and Dürr (2013), we would have obtained long steps and a joint grouping of middle and short steps. Thus, it is likely that middle and short steps were pooled together in previous publications. The short step class occurs less frequently in our analysis compared with Theunissen and Dürr (2013). We attribute this to the mentioned pooling in previous studies, but also to effects related to structural properties of the experimental setup. Paradigm-related effects on step parameters have been discussed for free walking as opposed to tethered walking on a treadwheel or a slippery plate (Cruse and Bartling, 1995) as well as for walkways of different slope (Grabowska et al., 2012). As Theunissen and Dürr (2013) worked with a narrow walkway, foot positions were generally close to the edges of the walkway, which could have increased the frequency of short tactile probing movements and/or correction steps to maintain stability (for review, see Dürr et al., 2018). In addition, middle steps, which were more frequent in our data, could have been less frequent on the narrow walkway, as these steps were often directed laterally and could be related to balancing, sideways translation and turning. Indeed, the turning-related increase in step-type asymmetry (Fig. 7) and the pronounced left–right asymmetry of middle step direction of inner and outer legs during guided turns (Fig. 8C) suggests that middle steps are an important means to control heading changes during turning. Similarly, the pronounced mediad steps of front legs observed in visual motion-induced turning (Dürr and Ebeling, 2005; Gruhn et al., 2009) correspond best to the step class which we, for now, have called middle steps.

That being said, the bimodal distribution of middle leg step direction (Fig. 7C) and the similarity of one of these modes with the prevalent long step direction (Fig. 7B) may indicate that our step-type classification is not ideal yet. Future work may need to include the complete limb kinematics to characterise middle steps in more detail. Similarly, a sequel study will focus on the occurrence of particular step types in temporal sequence and gait analysis, complementing our present understanding of middle step function.

### Visually guided versus spontaneous turns

Spontaneous turning in unrestrained terrestrial locomotion has been repeatedly studied in open-field settings without visual landmarks and little or no visual-motion cues (Strauss and Heisenberg, 1990; Jindrich and Full, 1999; Yang et al., 2024). Here, we used an open-field arena with a white wall (condition ‘all-white’) as a reference condition to address the questions: (i) do stick insects constrain their motor flexibility in a context-dependent manner, as indicated by differences compared with visually guided turns?; and (ii) do they compensate for the lack of visual guidance by increased tactile sampling, as indicated by more short steps?

We use the term ‘spontaneous’ to indicate that these turns must have been triggered intrinsically, rather than in response to some sort of timing or guiding stimulus. Note that animals were not visually deprived in the sense that there was no visual input at all (as would have been the case after blindfolding). Given the evidence for a contribution of negative phototaxis to stick insect visual orientation behaviour (Zeng et al., 2020; Meschenmoser and Dürr, 2025), spontaneous turns may have reflected an attempt to turn away from light. Even if this had been the case, the irregular initiation of turns and their wide range of curvatures suggest that the intrinsic drive to turn was neither continuous nor persistent. Alternative interpretations could be that spontaneous turns reflect ‘disorientation’ or an ‘intrinsic exploration behaviour’. In any case, they should be considered as non-guided, intrinsically triggered turns of variable curvature and with variable timing.

Spontaneous turns with a similar ROC were, on average, walked at lower speeds compared with visually guided turns (Fig. 6). At present, we cannot distinguish whether this is due to distinct turning strategies related to different initiation of turning or rather due to a difference in arousal or motivation related to the absence/presence of external stimuli. While the increased frequency of short steps for spontaneous turns with high curvature (Fig. 9) speaks for the former, the overall lower forward velocity (Fig. 6) speaks for the latter. Future work may need to test whether and how turning-related changes in parameters of long and middle steps depend on forward velocity in the same way in visually guided and spontaneous turns. As all-white trials were, on average, walked at lower speed, it is possible that the primary effect of a lack of visual guidance is the increase in curvature, in turn leading to a reduction in forward velocity. Our finding that short steps were more frequent in narrow curves only (Fig. 9) supports the interpretation that short steps serve as correction steps (Theunissen and Dürr, 2013), as static stability is increasingly compromised at tighter turns.

### Conclusion

Taken together, our results show that stick insects alter their turning behaviour depending on the visual context, as revealed by a reduced overall speed and increased frequency of short steps. When turning towards static visual targets, turning responses scaled non-linearly, where scaling was dominated by an increase in turning duration rather than maximum rotational velocity. Increased rotational velocity corresponded with reduced forward velocity. These changes were caused by larger shifts in step direction, as well as an increased asymmetry in step types between inner and outer legs, with an increased frequency of middle steps on the inner side of the curve. In summary, our results suggest a mix of distinct turning strategies with both the scaling of step parameters and the likelihood of a particular step class depending on overall turn angle.

## Supplementary Material

10.1242/jexbio.252608_sup1 Supplementary information
