# Supplementary material for: Walking in circles: linking high- and low-level parameter scaling of visually guided and spontaneous turning behaviour
Source: J Exp Biol. 2026 Oct 6;229(19):jeb252608. doi: 10.1242/jeb.252608 (PMC13723402; doi:10.1242/jeb.252608)
Supplement: Supplementary file 1 [file jexbio-229-252608-s1.pdf]

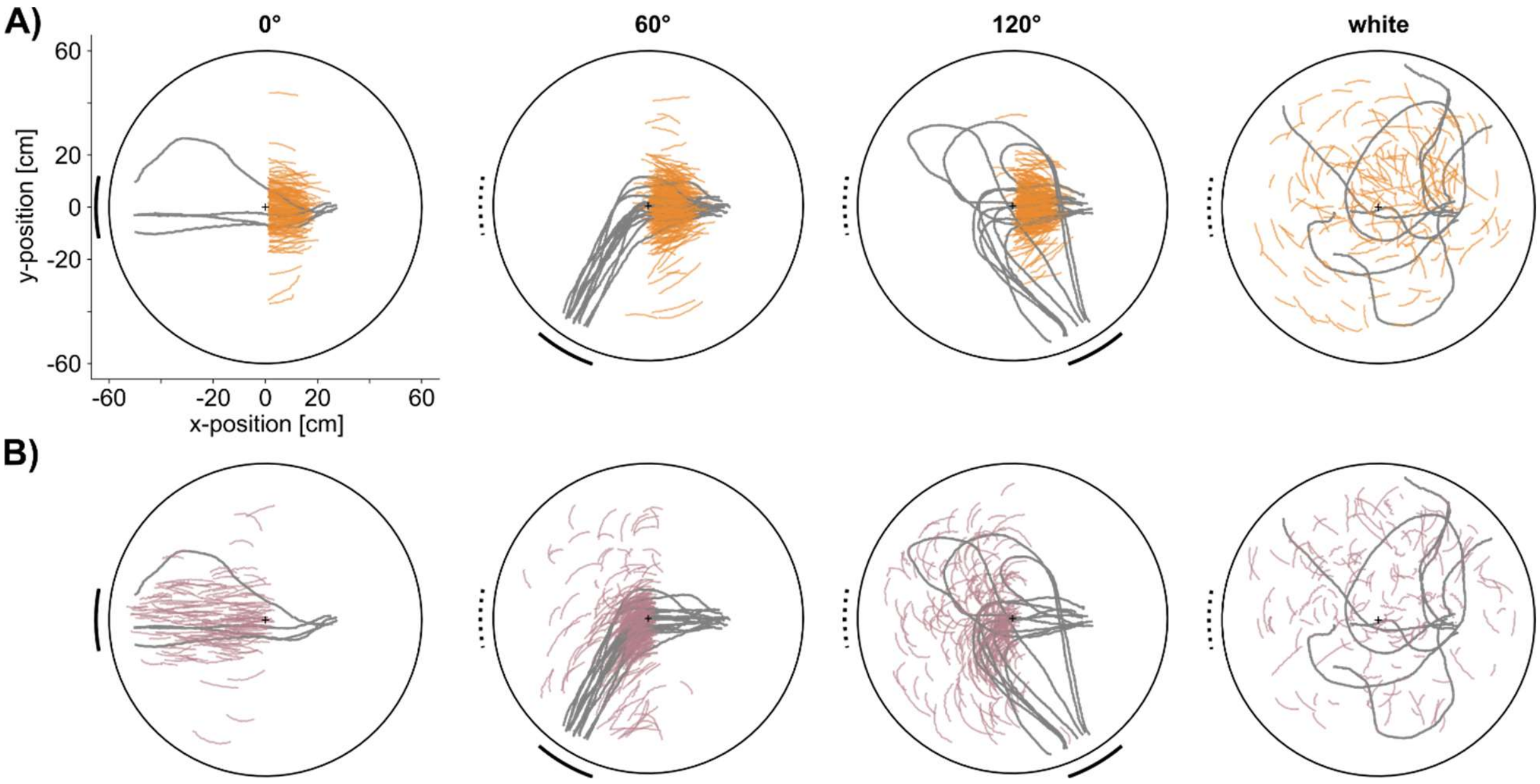

Fig. S1. Example trials and analysis episodes A and B. Top view of the circular arena. Coloured trajectory parts show the prothorax position of '*on target*' trials. Each column corresponds to one of the four conditions, where 0°, 60° and 120° are "landmark trials" with (60°, 120°) and without displacement (0°) and "white" refers to the all-white reference condition without landmarks. Continuous underlying grey lines show exemplary trials of one randomly chosen animal. (**A**) In landmark trials, episode A started three seconds before the displacement of the black bar in 60° and 120° trials, or three seconds before crossing of the midline = control trials without displacement (0°). In all-white trials, it started half-way into the trial duration. (**B**) Episode B spanned the three-second period with largest mean rotation, after episode A.

Journal of Experimental Biology • Supplementary information

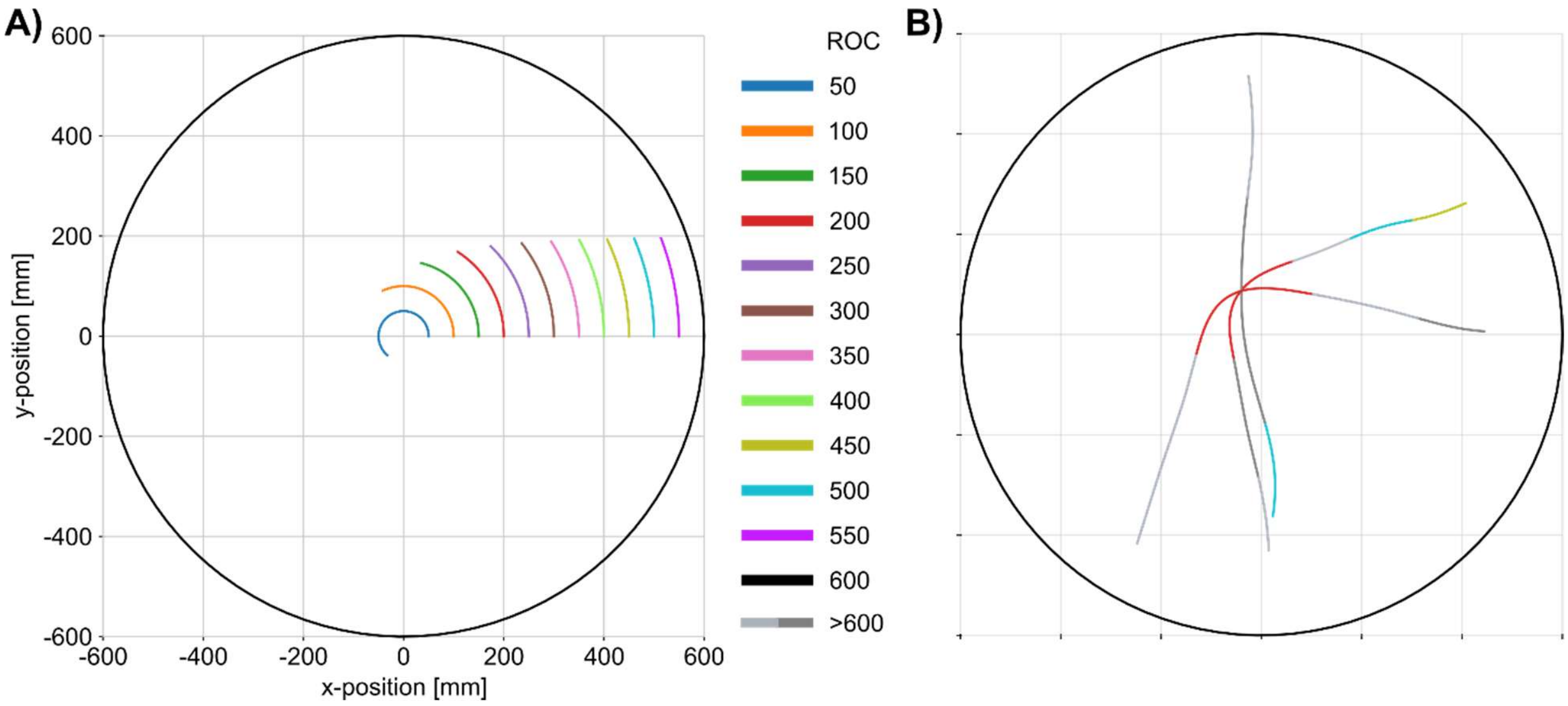

Fig. S2. Trajectory segmentation according to curvature. (**A**) Schematic illustration of segments of the same length with different radius of curvature (ROC) in mm. The outer black circle shows the arena size (radius: 600 mm). (**B**) Segmented trajectories of three exemplary trials. Segments are coloured according to their ROC. Legend in (A) lists upper bounds of ROC ranges. For example, red segments have a ROC between 200 and 249 mm. Different grey segments have a ROC larger than 600 mm.

Journal of Experimental Biology • Supplementary information

Table S1. p-values of a-priori post-hoc tests on matched pairs (Wilcoxon's signed rank test) for 'at wall' and 'on target' trials shown in Fig. 4C.

**Comparison**	**At wall**	**On target**
**Landmark vs. all-white**	<0.0001	n.a.
**Starting orientation**	0.17	0.92
**Displacement vs. stationary**	0.63	0.73
**Displacement magnitude**	<0.0001	<0.0001
**Side of displacement**	0.6	0.23

Table S2. Friedman test for matched samples results for differences in path length, curvature, median and maximum forward velocity, shown in Fig. 4D. If the Friedman test was statistically significant, Bonferroni-corrected Wilcoxon's signed-rank post-hoc tests for were done (α_{corr} = 0.05/6 = 0.0083). Mean and standard error of the mean are given for each condition.

	**Path length [mm]**		**Curvature**		**Median velocity [mm/s]**		**Maximal velocity [mm/s]**	
**comparison**	Friedman	Wilcoxon	Friedman	Wilcoxon	Friedman	Wilcoxon	Friedman	Wilcoxon
	<0.0001		<0.0001		<0.0001		0.36	
**120 vs. 60**		<0.0001		<0.0001		0.006		
**120 vs. 0**		<0.0001		<0.0001		<0.0001		
**120 vs. white**		0.015		<0.0001		<0.0001		
**0 vs. white**		<0.0001		<0.0001		<0.0001		
**60 vs. 0**		<0.0001		<0.0001		0.347		
**60 vs. white**		<0.0001		<0.0001		<0.0001		
	**mean [mm]**	**s.e.m. [mm]**	**mean**	**s.e.m.**	**mean [mm/s]**	**s.e.m. [mm/s]**	**mean [mm/s]**	**s.e.m. [mm/s]**
**0**	987.33	28.81	1.31	0.04	39.66	1.17	142.64	2.73
**60**	1121.45	39.33	1.63	0.05	38.58	1.07	145.38	2.52
**120**	1465.30	36.75	3.07	0.09	34.67	1.07	143.48	2.89
**white**	1765.23	99.95	6.50	0.79	29.16	1.19	141.26	2.27

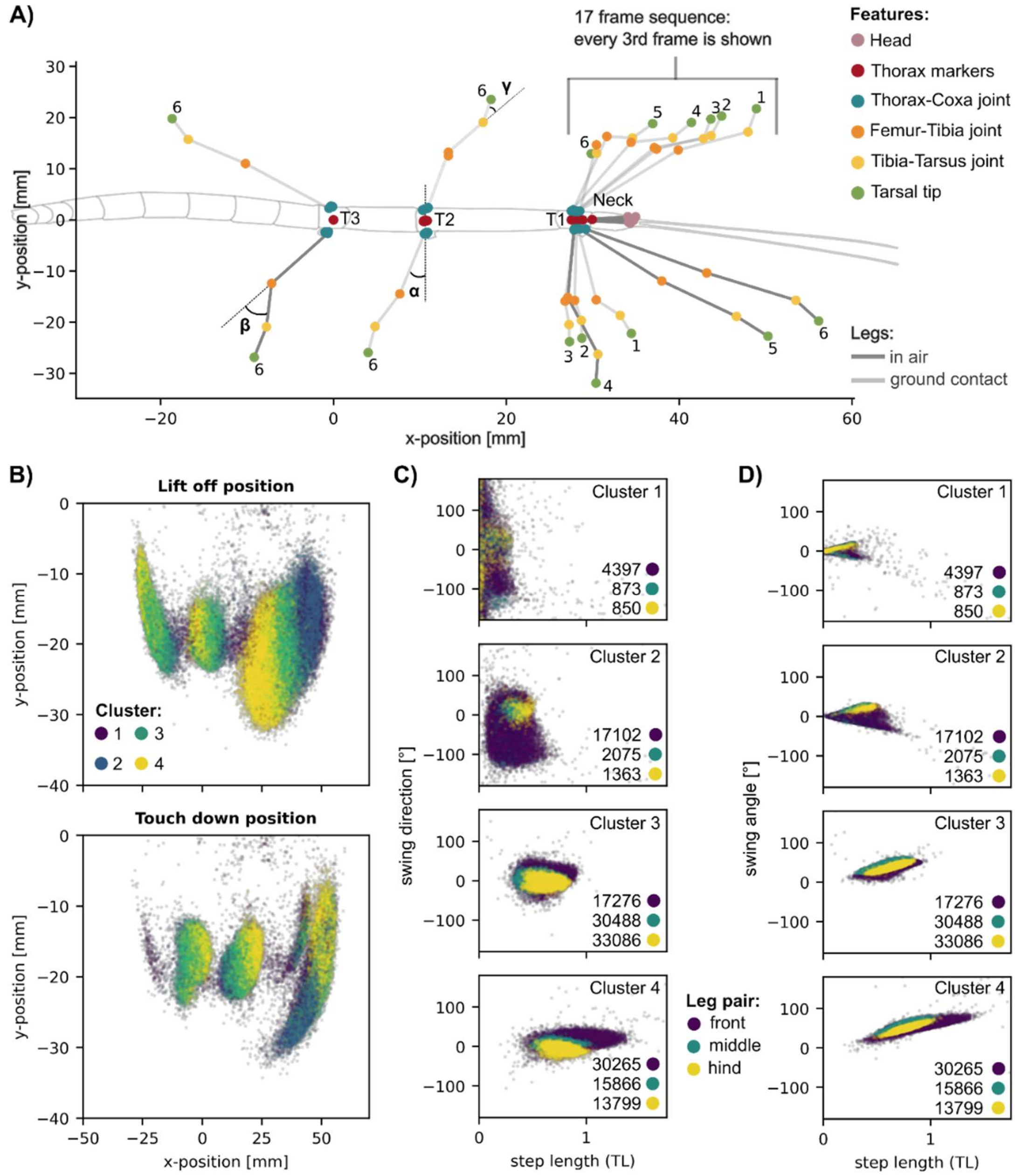

Fig. S3. Classification of step cycle phase and type of step. (**A**) Classification of step cycle phase, distinguishing episodes "with ground contact" (stance) from episodes "with no ground contact" (swing). Schematic drawing of a stick insect with all tracked features, including exemplary results for stance in the left front leg and for swing in the right front leg. The three leg angles, α, β and γ are shown for right middle, right hind and left middle legs, respectively, but were calculated for all legs. Legs drawn in dark grey were classified as 'with no ground contact' (right front and hind legs), those drawn in light grey were classified as 'with ground contact'. For front legs, one leg every third frame was plo ed from a temporal sequence of 17 frames. Numbers besides the legs indicate the temporal succession. **(B-D)** Step type classifica on. **(B)** Li -off and touch-down posi on of four different clusters of steps for three leg pairs. Le legs were mirrored for plo ng. Posi ons were centred on the metathorax marker T3. **(C)** Swing direc on is plo ed against step length for four different clusters; colours show different leg pairs. Numbers within each subplot show the number of steps per cluster and leg pair. **(D)** Swing angle is plo ed against step length for four different clusters, with details as in C.

Journal of Experimental Biology • Supplementary information

Table S3. Results of Friedman tests for matched samples for differences in percentage of three classes of steps between straight and curve walking, and between inner and outer legs, shown in Fig. 7. If the Friedman test was statistically significant, Bonferroni-corrected Wilcoxon's signed-rank post-hoc tests on pairwise comparisons were done (α_{corr} = 0.05/3 = 0.0167). Mean and standard error of the mean are given for each condition.

	**Inner leg**		**Outer leg**	
**comparison**	Friedman<0.0001		Friedman<0.0001	
**curve vs. straight, long steps**	p<0.0001		p<0.0001	
**curve vs. straight, middle steps**	p<0.0001		p<0.0001	
**curve vs. straight, short steps**	p<0.0001		p<0.0001	
	**Straight**		**Curve**	
**comparison**	Friedman<0.0001		Friedman<0.0001	
**inner vs. outer, long steps**	p=0.0003		p<0.0001	
**inner vs. outer, middle steps**	p=0.0006		p<0.0001	
**inner vs. outer, short steps**	p=0.44		p=0.03	
	**Inner leg**		**Outer leg**	
	**mean [%]**	**s.e.m. [%]**	**mean [%]**	**s.e.m. [%]**
**curve long**	59.42	± 2.32	77.98	± 1.78
**curve middle**	33.51	± 1.99	16.05	± 1.36
**curve short**	7.07	± 1.3	5.97	± 0.84
**straight long**	74.56	± 1.84	77.22	± 1.74
**straight middle**	20.45	± 1.52	17.84	± 1.53
**straight short**	4.99	± 0.9	4.94	± 0.84

Journal of Experimental Biology • Supplementary information

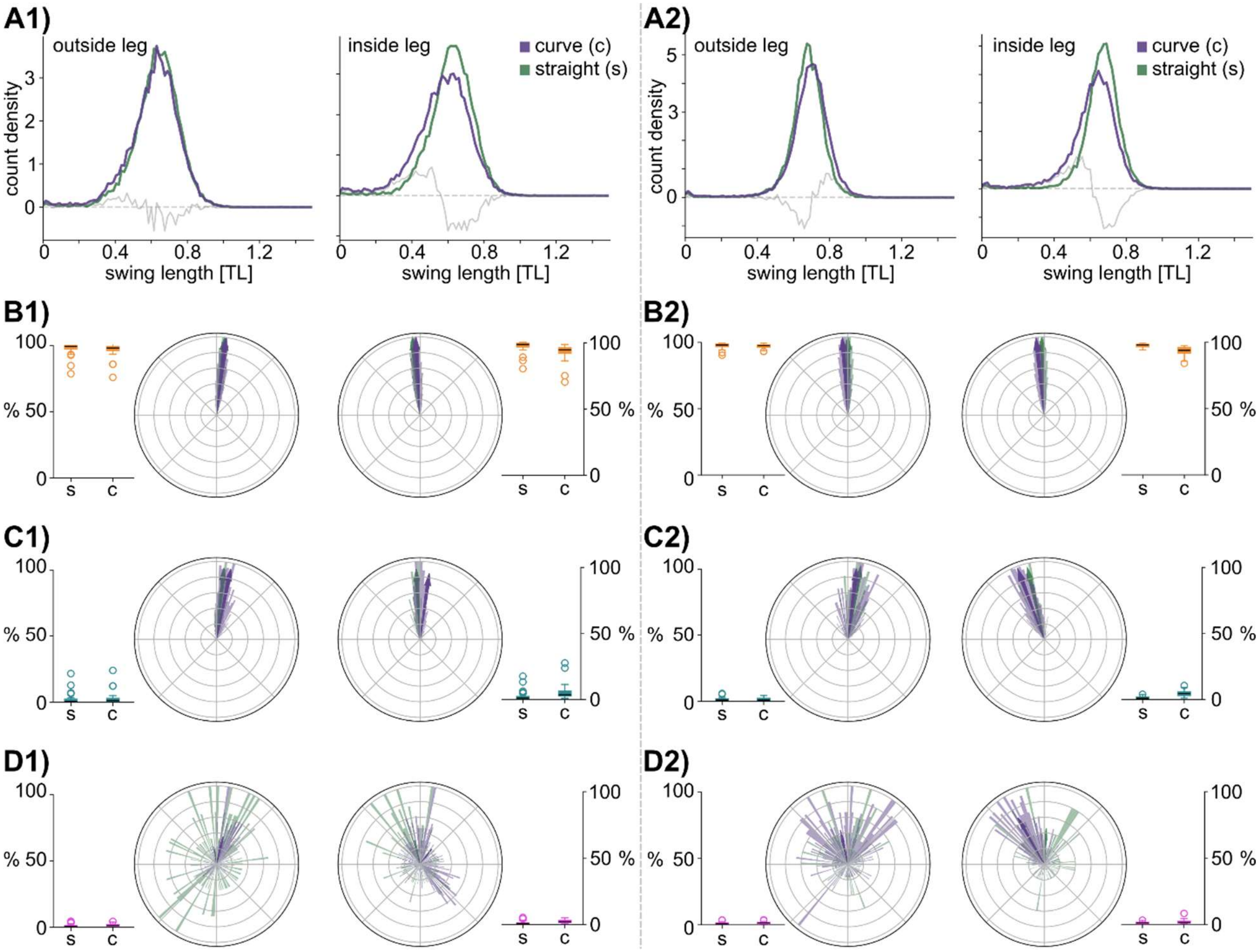

Fig. S4. Three classes of step types in middle and hind legs. Same graph details as in Fig. 7 of main manuscript, but for different leg types. (**A**) Density plots showing the distribution of swing lengths of middle (1) and hind (2) legs (left: outer leg, right: inner leg) in curved (purple, ROC <= 600, N=32, Middle legs: n_{steps_inside}= 15706, $n_{steps\_outside}$= 16919; Hind legs: n_{steps_inside}= 15570, $n_{steps\_outside}$= 16812) and straight segments (turquoise, ROC > 600, N=32, Middle legs: n_{steps_inside} = 12200, $n_{steps\_outside}$ = 12263; Hind legs: n_{steps_inside} = 12190, $n_{steps\_outside}$ = 12291). The grey solid line shows the difference between the curved and straight condition. The grey dashed line highlights zero density. N = 32 (**B-D**) Circular histograms showing the swing direction of steps of front legs (left column: outside leg, right column: inside leg) in curved (purple) and straight (green) segments. The mean direction (dir) and the concentration around mean direction (r) are given below. Boxplots show the percentage of a step type per condition as means per animal. N = 31. (**B**) Long steps. (**C**) Middle steps. (**D**) Short steps.

Journal of Experimental Biology • Supplementary information

Table S4. Results of Friedman tests for matched samples for differences in overall turn angle (Fig. 8A) and step type asymmetry (Fig. 8B). If the Friedman test was statistically significant, Bonferroni-corrected Wilcoxon's signed-rank post-hoc tests on paired comparisons were done (α_{corr} = 0.05/4 = 0.0125). Mean and standard error of the mean are given for each condition. e. A: episode A; e. B: episode B.

	**Rotation [°]**		**Asymmetry**	
	Friedman p<0.0001		Friedman p<0.0001	
**60 e. A vs. 120 e. A**	p = 0.9		p = 0.02	
**60 e. B vs.120 e. B**	p<0.0001		p = 0.0015	
**60 e. A vs. 60 e. B**	p<0.0001		p<0.0001	
**120 e. A vs. 120 e. B**	p<0.0001		p<0.0001	
	**mean [°]**	**s.e.m. [°]**	**mean**	**s.e.m.**
**60 e. A**	11.42	0.81	0.99	0
**60 e. B**	57.56	0.98	0.88	0.02
**120 e. A**	11.4	1.2	0.97	0.01
**120 e. B**	88.28	2.46	0.73	0.04

Journal of Experimental Biology • Supplementary information

Table S5. Results of Friedman tests for matched samples for differences in change of swing direction of long steps between episode A and B, shown in Fig. 8C2. If the Friedman test was statistically significant, Bonferroni-corrected Wilcoxon's signed-rank post-hoc tests on paired comparisons were done (α_{corr} = 0.05/4 = 0.0125). Mean and standard error of the mean are given for each condition.

	**Direction**		**Variance**	
	Friedman p<0.0001		Friedman p<0.0001	
**60 inside vs. 120 inside**	p=0.0048		p=0.2941	
**60 outside vs.120 outside**	p=0.0001		p=0.0534	
**60 inside vs. 60 outside**	p=0.3579		p<0.0001	
**120 inside vs. 120 outside**	p=0.7172		p<0.0001	
	**mean**	**s.e.m.**	**mean**	**s.e.m.**
**60 inside**	7.07	0.63	0.0243	0.0033
**60 outside**	7.91	0.61	-0.0007	0.0014
**120 inside**	14.7	2.62	0.0226	0.0071
**120 outside**	14.38	1.24	-0.0062	0.0022

Table S6. Results of Friedman tests for matched samples for differences in change of swing direction of middle steps between episode A and B, shown in Fig. 8C3. Mean and standard error of the mean are given for each condition below.

	**Direction**		**Variance**	
	Friedman p=0.05		Friedman p=0.22	
	**mean**	**s.e.m.**	**mean**	**s.e.m.**
**60 inside**	30.17	9.45	0.0881	0.0411
**60 outside**	-17.54	14.59	-0.0137	0.0627
**120 inside**	28.43	12.44	0.1401	0.0615
**120 outside**	2.19	10.87	0.0858	0.0868

Table S7. Friedman test for matched samples results for differences in percentages of short steps for different ROCs, shown in Fig. 9. Bonferroni-corrected Wilcoxon's signed-rank post-hoc tests were done for paired comparisons (α_{corr} = 0.05/4 = 0.0125). Mean and standard error of the mean are given for each condition on the left.

	**mean [%]**	**s.e.m. [%]**		Friedman p<0.0001
**1-200 white**	6.08	0.73		**Wilcoxon:**
**201-400 white**	3.79	0.94	**white vs stim. (1-200)**	p=0.0024
**401-600 white**	4.12	0.77	**white vs stim. (201-400)**	p=0.9205
**601-4000 white**	3.84	0.66	**white vs stim. (401-600)**	p=0.7835
**1-200 stim**	4.6	0.57	**white vs stim. (601-4000)**	p=0.0385
**201-400 stim**	3.48	0.55		
**401-600 stim**	3.49	0.57		
**601-4000 stim**	2.73	0.42		

Journal of Experimental Biology • Supplementary information

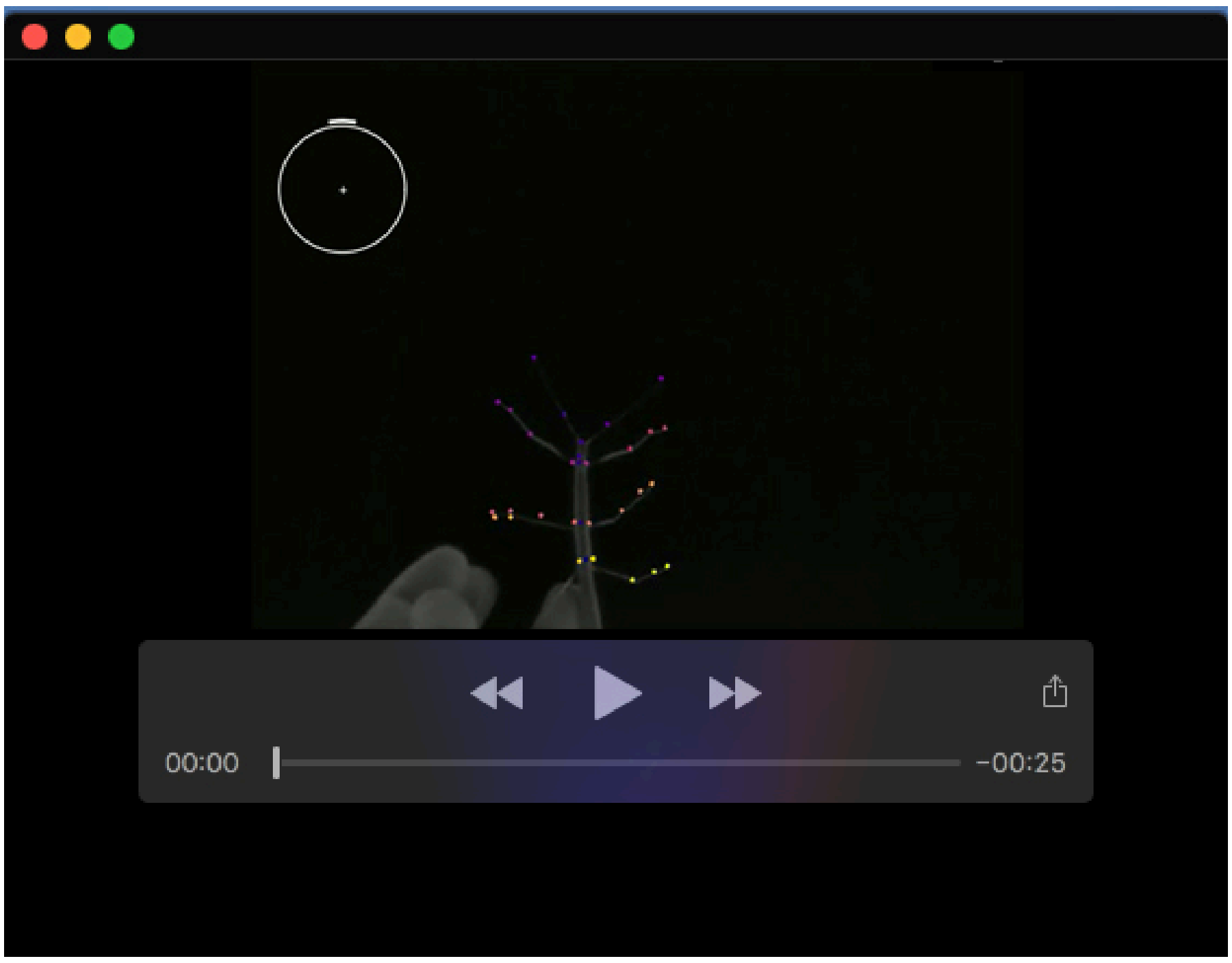

Movie 1. Example trial of animal walking through the arena in a 0° trial. Coloured points show marker-positions obtained from *DeepLabCut* (DLC) tracking. Upper left white plot shows circular arena with black bar, prothorax position as well as front leg tibia-tarsus joint positions throughout the trial. Arrows show swing direction of right (cyan) and left (red) front legs respectively.

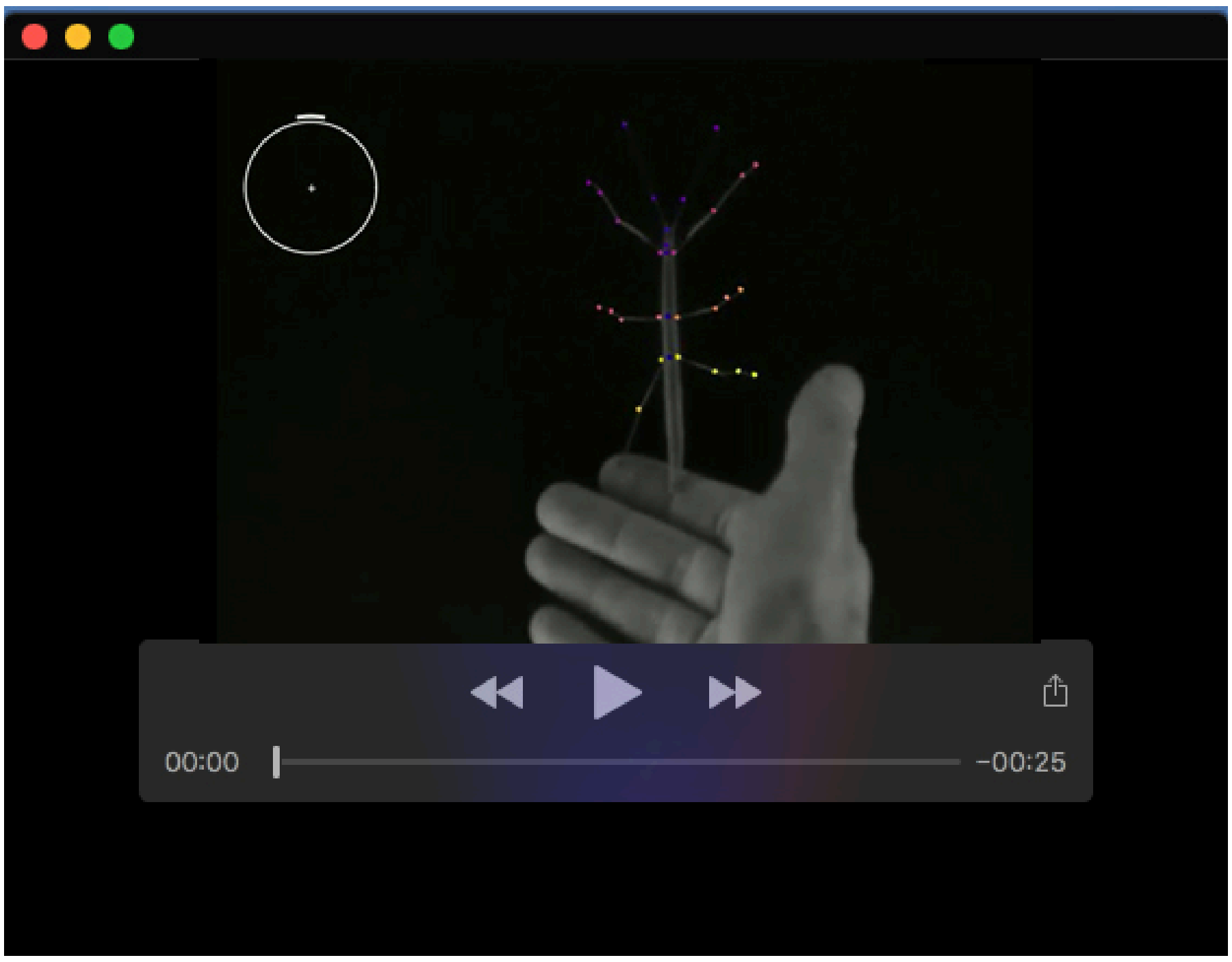

Movie 2. Example trial of animal walking through the arena in a 60° trial. Coloured points show marker-positions obtained from DLC tracking. Upper left white plot shows circular arena with black bar, prothorax position as well as front leg tibia-tarsus joint positions throughout the trial. Arrows show swing direction of right (cyan) and left (red) front legs respectively.

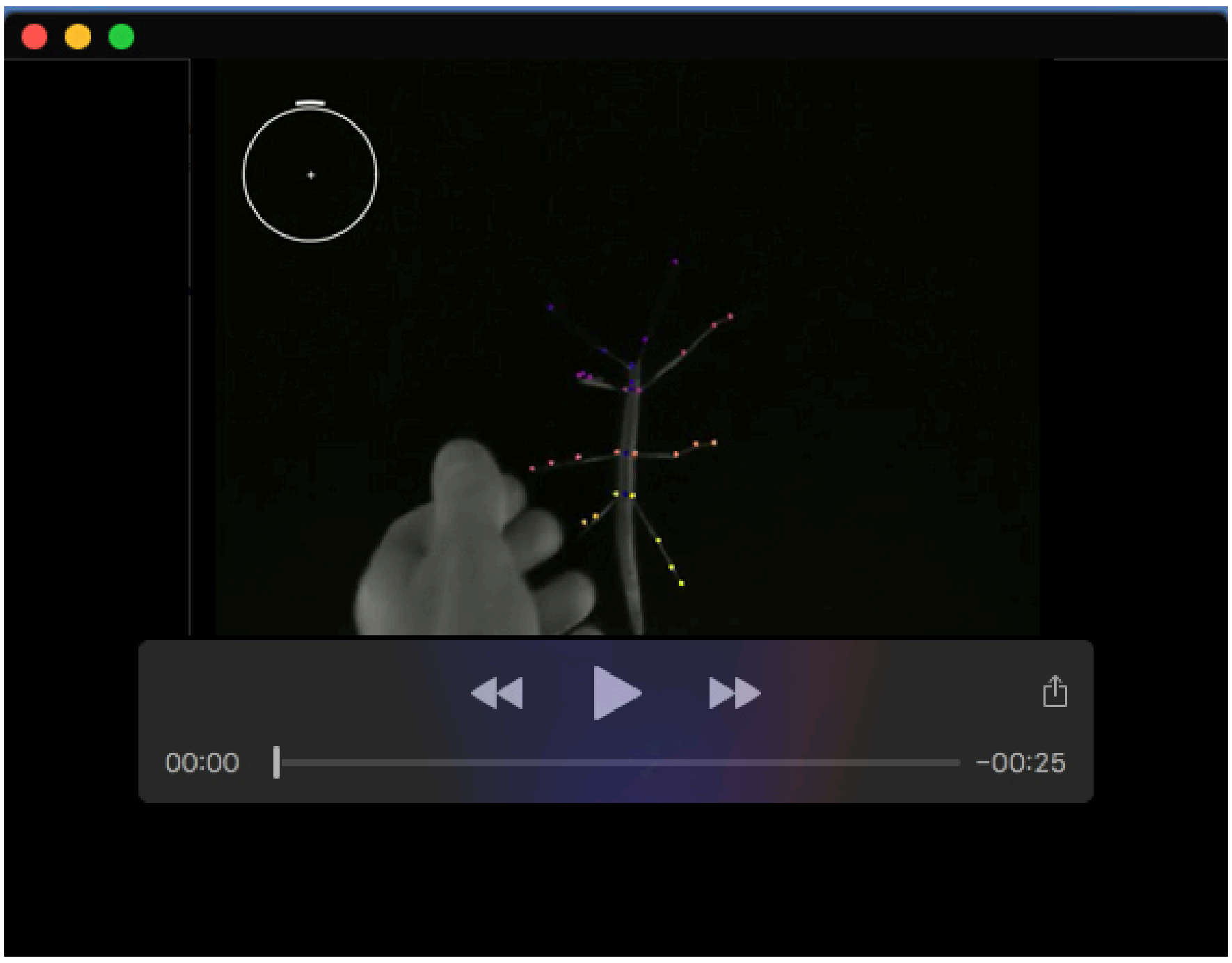

Movie 3. Example trial of animal walking through the arena in a 120° trial. Coloured points show marker-positions obtained from DLC tracking. Upper left white plot shows circular arena with black bar, prothorax position as well as front leg tibia-tarsus joint positions throughout the trial. Arrows show swing direction of right (cyan) and left (red) front legs respectively.

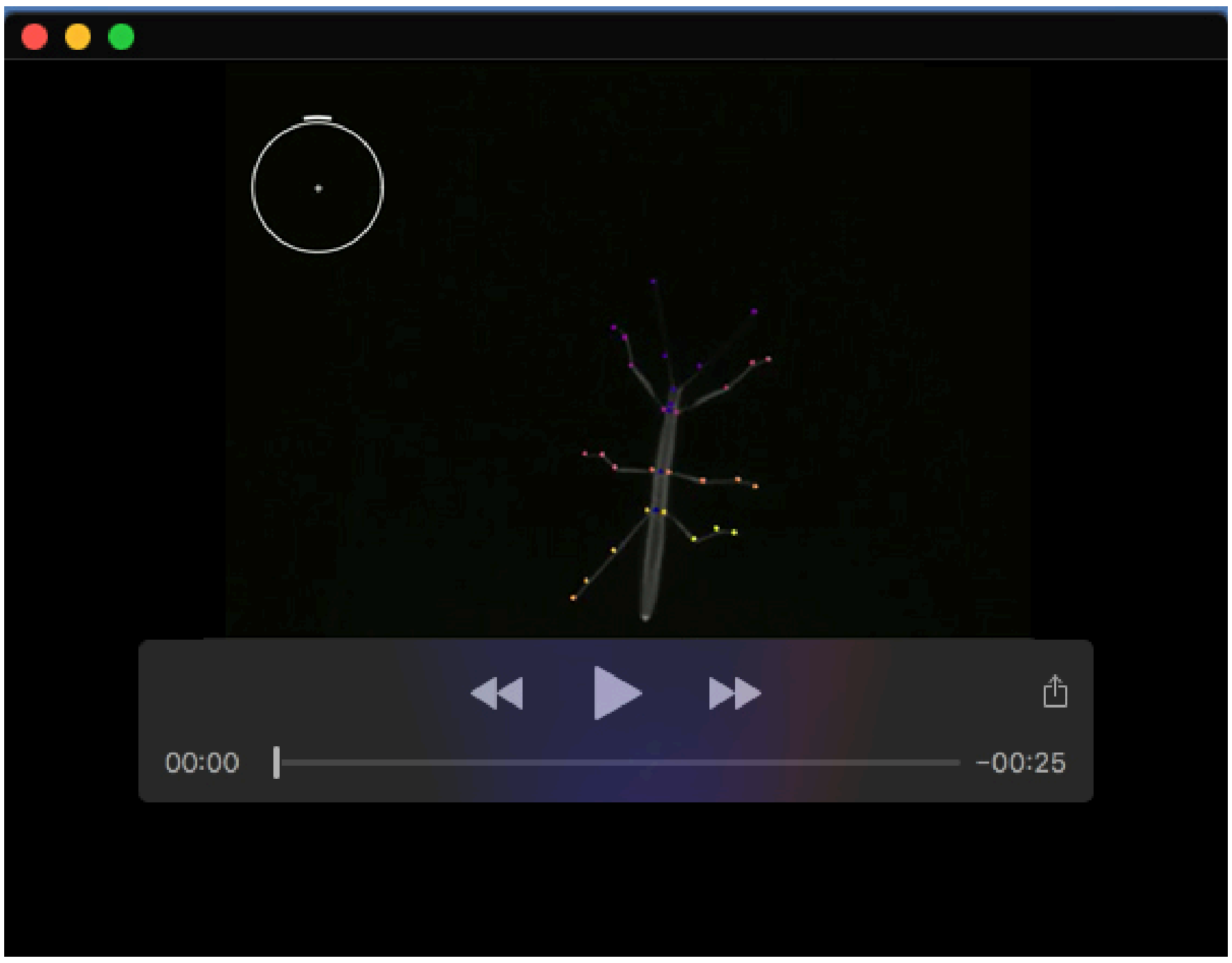

Movie 4. Example trial of animal walking through the arena in an all-white trial. Coloured points show marker-positions obtained from DLC tracking. Upper left white plot shows circular arena, prothorax position as well as front leg tibia-tarsus joint positions throughout the trial. Note that the bar was not present in this trial, and is shown in the insert only to indicate the starting orientation of the trial. Arrows show swing direction of right (cyan) and left (red) front legs respectively.

Journal of Experimental Biology • Supplementary information
